# Supplementary material for: Prebiotic condensation through wet–dry cycling regulated by deliquescence
Source: Nat Commun. 2019 Oct 4;10:4508. doi: 10.1038/s41467-019-11834-1 (PMC6778215; doi:10.1038/s41467-019-11834-1)
Supplement: Supplementary file 1 — Supplementary Information [file 41467_2019_11834_MOESM1_ESM.pdf]

Supplementary Information

Prebiotic Condensation Through Wet–Dry Cycling Regulated by Deliquescence

Thomas D. Campbell[1,†], Rio Febrian[1,†], Jack T. McCarthy[1], Holly E. Kleinschmidt[1], Jay G. Forsythe[2], and Paul J. Bracher[1,*]

[1] Department of Chemistry, Saint Louis University, 3501 Laclede Avenue, St. Louis, Missouri 63103 USA

[2] Department of Chemistry and Biochemistry, College of Charleston, 66 George Street, Charleston, South Carolina 29424 USA

[†] These authors contributed equally to this work.

[*] Corresponding Author. E-mail: paul.bracher@slu.edu

Table of Contents

Supplementary Figures 4
Supplementary Figure 1 | Photograph of various pure salts of Na^+ and K^+. 4
Supplementary Figure 2 | Demonstration of a deliquescent mixture reaching equilibrium. 5
Supplementary Figure 3 | The deliquescence-lowering property of chloride mixtures. 5
Supplementary Figure 4 | Oligomer distribution in chloride mixtures 70 %RH 120 °C. 6
Supplementary Figure 5 | Oligomer distribution in chloride mixtures 70 %RH 100 °C. 6
Supplementary Figure 6 | Oligomer distribution in chloride mixtures 50 %RH 120 °C. 7
Supplementary Figure 7 | Oligomer distribution in chloride mixtures 50 %RH 100 °C. 7
Supplementary Figure 8 | Oligomer distribution in chloride mixtures 30 %RH 120 °C. 8
Supplementary Figure 9 | Oligomer distribution in chloride mixtures 30 %RH 100 °C. 8
Supplementary Figure 10 | Oligomer distribution in K_2HPO_4 mixtures 50 %RH 120 °C. 9
Supplementary Figure 11 | Oligomer distribution in Na_2HPO_4 mixtures 50 %RH 120 °C. 9
Supplementary Figure 12 | Solution-start yield comparison in chloride samples. 10
Supplementary Figure 13 | Oligomer distribution in chloride solution 70 %RH 100 °C. 11
Supplementary Figure 14 | Oligomer distribution in chloride solution 30 %RH 100 °C. 11
Supplementary Figure 15 | Solution-start yield comparison in dibasic phosphate samples. 12
Supplementary Figure 16 | Oligomer distribution in K_2HPO_4 solution 50 %RH 100 °C. 13
Supplementary Figure 17 | Oligomer distribution in Na_2HPO_4 solution 50 %RH 100 °C. 13
Supplementary Figure 18 | Oligomer distribution in K_2HPO_4 solution 50 %RH 120 °C. 14
Supplementary Figure 19 | Oligomer distribution in Na_2HPO_4 solution 50 %RH 120 °C. 14
Supplementary Figure 20 | Yield comparison in monobasic phosphate samples. 15
Supplementary Figure 21 | Oligomer distribution in KH_2PO_4 mixtures 70 %RH 100 °C. 16
Supplementary Figure 22 | Oligomer distribution in NaH_2PO_4 mixtures 70 %RH 100 °C. 16
Supplementary Figure 23 | Yield comparison in bromide samples. 17
Supplementary Figure 24 | Oligomer distribution in KBr mixtures 50 %RH 100 °C. 18
Supplementary Figure 25 | Oligomer distribution in NaBr mixtures 50 %RH 100 °C. 18
Supplementary Figure 26 | Oligomer distribution in KBr mixtures 70 %RH 100 °C. 19
Supplementary Figure 27 | Oligomer distribution in KBr mixtures 70 %RH 100 °C. 19
Supplementary Figure 28 | Example chromatograms of glycine oligomerization. 20
Supplementary Figure 29 | Chromatogram of the MALDI sample. 21
Supplementary Figure 30 | MALDI–TOF data. 22
Supplementary Figure 31 | Yield comparison of various hydration models. 23
Supplementary Figure 32 | Brine overhydration samples. 24
Supplementary Tables 25
Supplementary Table 1 | Summary of the glycine wet–dry cycling with various salts. 25
Supplementary Table 2 | Summary of the glycine solution-start wet–dry cycling. 26
Supplementary Table 3 | Summary of the data shown in Supplementary Figure 1. 26

Supplementary Table 4 | Summary of the data shown in Supplementary Figure 4. 27
Supplementary Table 5 | Summary of the data shown in Supplementary Figure 5. 27
Supplementary Table 6 | Summary of the data shown in Supplementary Figure 6. 28
Supplementary Table 7 | Summary of the data shown in Supplementary Figure 7. 28
Supplementary Table 8 | Summary of the data shown in Supplementary Figure 8. 29
Supplementary Table 9 | Summary of the data shown in Supplementary Figure 9. 29
Supplementary Table 10 | Summary of the data shown in Supplementary Figure 10. 30
Supplementary Table 11 | Summary of the data shown in Supplementary Figure 11. 30
Supplementary Table 12 | Summary of the data shown in Supplementary Figure 13. 31
Supplementary Table 13 | Summary of the data shown in Supplementary Figure 14. 31
Supplementary Table 14 | Summary of the data shown in Supplementary Figure 16. 32
Supplementary Table 15 | Summary of the data shown in Supplementary Figure 17. 32
Supplementary Table 16 | Summary of the data shown in Supplementary Figure 18. 33
Supplementary Table 17 | Summary of the data shown in Supplementary Figure 19. 33
Supplementary Table 18 | Summary of the data shown in Supplementary Figure 21. 34
Supplementary Table 19 | Summary of the data shown in Supplementary Figure 22. 34
Supplementary Table 20 | Summary of the data shown in Supplementary Figure 24. 34
Supplementary Table 21 | Summary of the data shown in Supplementary Figure 25. 35
Supplementary Table 22 | Summary of the data shown in Supplementary Figure 26. 35
Supplementary Table 23 | Summary of the data shown in Supplementary Figure 27. 36
Supplementary Table 24 | MALDI data of glycine oligomerization reaction after cycle 15. 36
Supplementary Table 25 | ICP–OES data. 37
Supplementary Methods 37
Supplementary Discussion 43
Supplementary References 48

Supplementary Figures

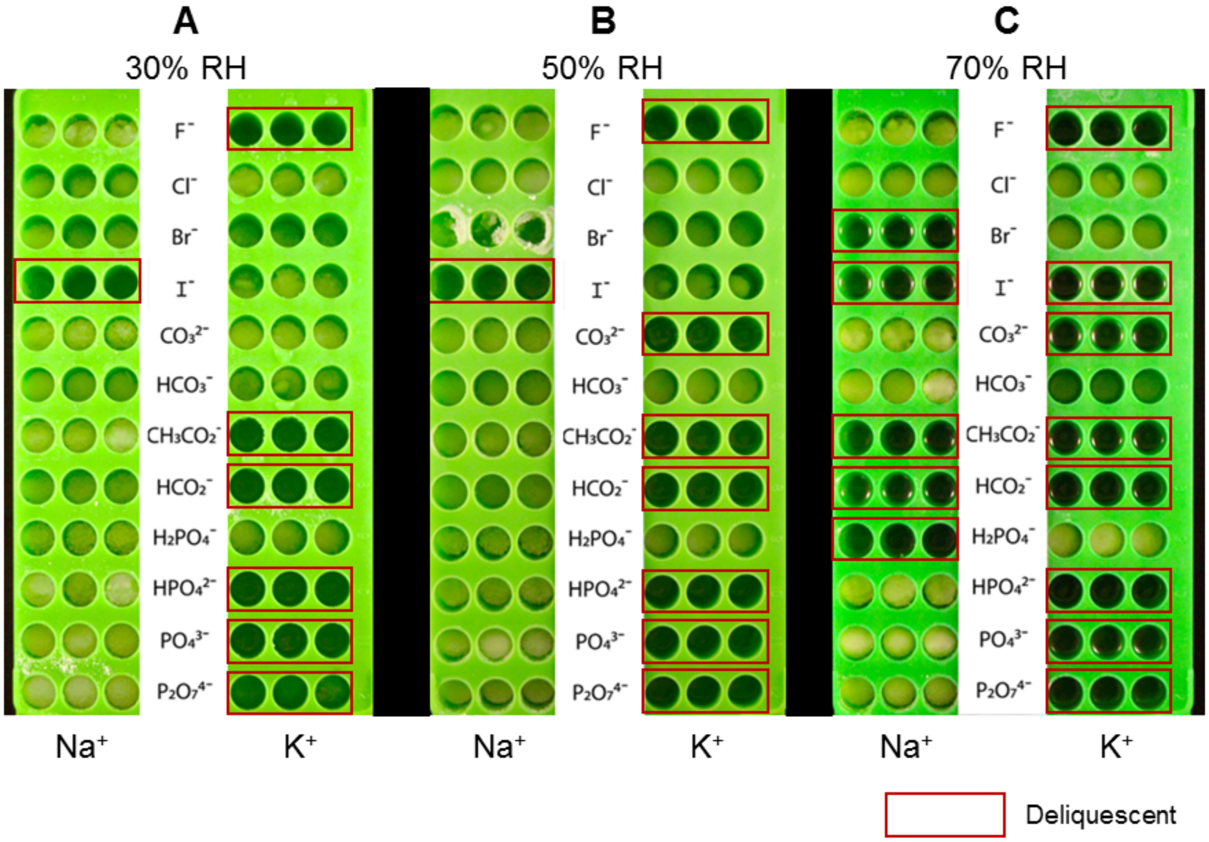

Supplementary Figure 1 | Photograph of various pure salts of Na^+ and K^+. Photo was taken after 28 days of exposure to 40 °C and 30% RH (A), 50% RH (B), and 70% RH (C). A solid white sample of each reagent grade salt was placed in three wells and the trays placed in temperature and humidity-controlled chambers for 28 days. Each tray was exposed to a different relative humidity at 40 °C. Each row contains three samples of the sodium salt (left) of the indicated counteranion and three samples of the potassium salt (right). Red boxes identify deliquescent samples which absorbed water vapor to form homogenous aqueous solutions. The unboxed samples remained solid.

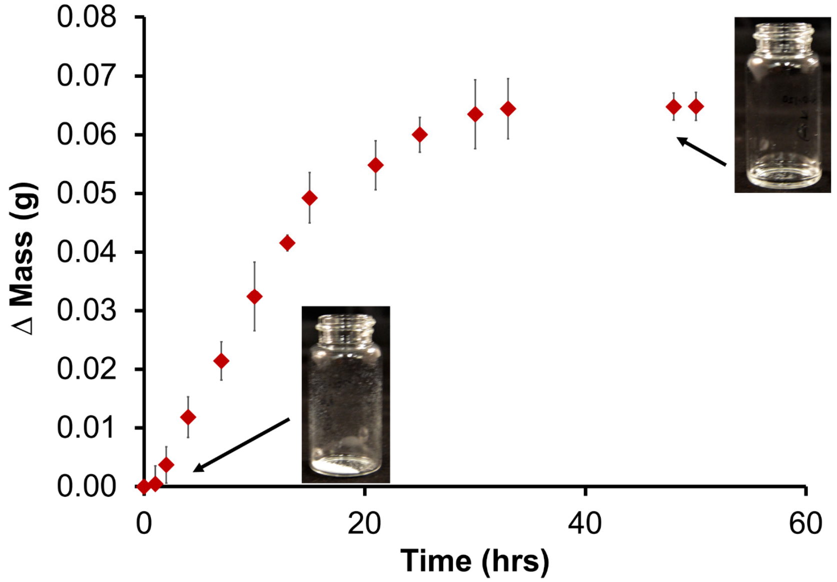

Supplementary Figure 2 | Demonstration of a deliquescent mixture reaching equilibrium. The change in mass for a representative sample of the mixture of glycine with potassium and sodium salts of chloride and hydroxide (K^+,Na^+)(^-Cl,^-OH) and glycine mixture at 40 °C and 70 %RH over time. This plot demonstrates that after 20 hours, the sample has collected approximately 85% of the maximum volume it would collect if it were allowed to reach equilibrium. Error bars represent 95% confidence intervals (n=3 identical experiments). Source data are provided as a Source Data file.

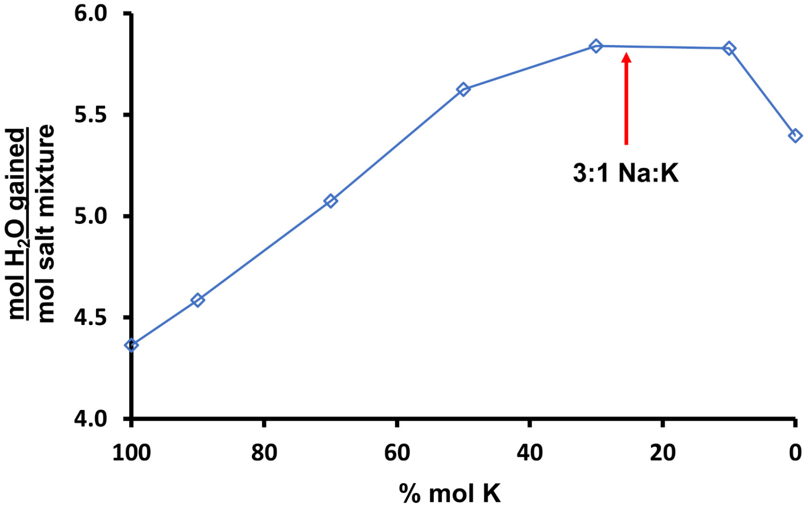

Supplementary Figure 3 | The deliquescence-lowering property of chloride mixtures. Various mixtures of glycine with potassium and sodium salts of chloride and hydroxide (K^+,Na^+)(^-Cl,^-OH) exposed to 40 °C and 70 %RH after 24 hours. Source data are provided as a Source Data file.

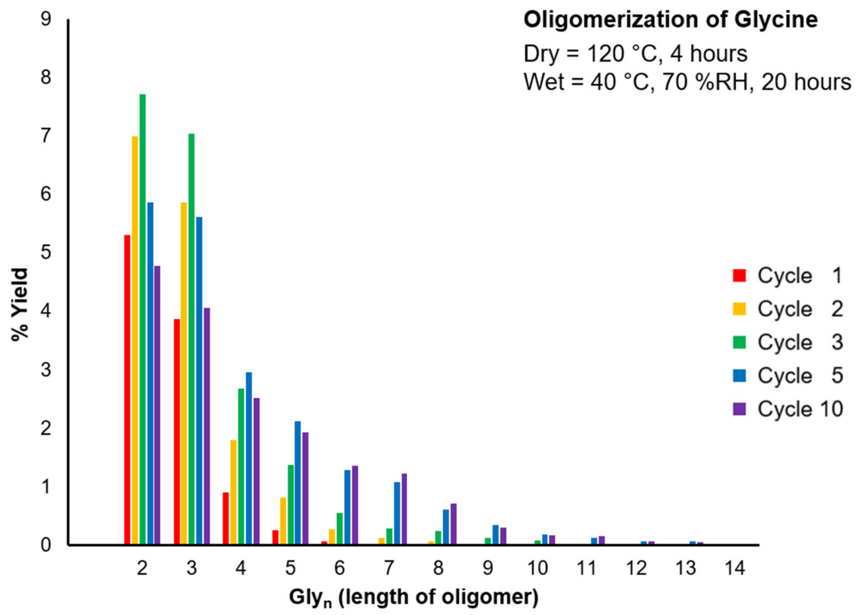

Supplementary Figure 4 | Oligomer distribution in chloride mixtures 70 %RH 120 °C. Distribution of oligomer length after cycles 1, 2, 3, 5, and 10 in the samples prepared with (K^+,Na^+)(^-Cl,^-OH). Each cycle was 20 hours at 40 °C and 70 %RH followed by 4 hours at 120 °C. Source data are provided as a Source Data file.

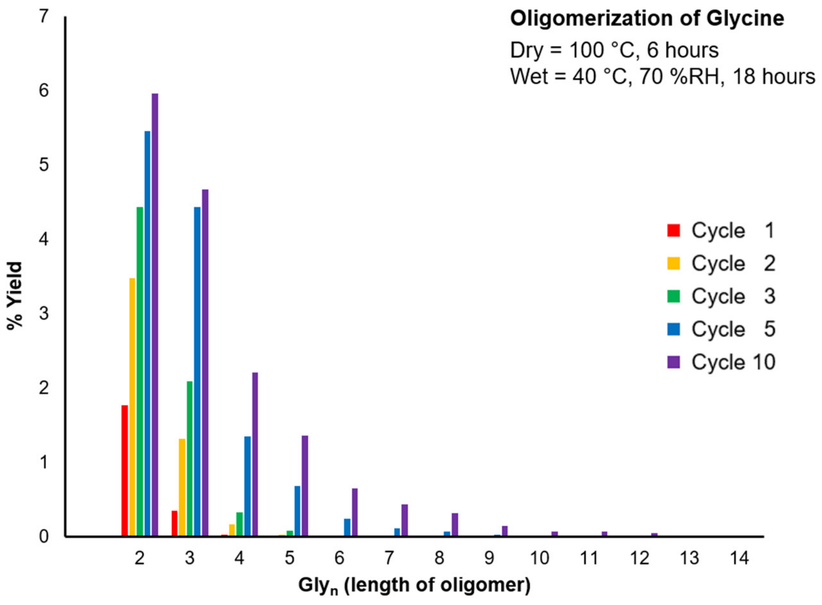

Supplementary Figure 5 | Oligomer distribution in chloride mixtures 70 %RH 100 °C. Distribution of oligomer length after cycles 1, 2, 3, 5, and 10 in the samples prepared with (K^+,Na^+)(^-Cl,^-OH). Each cycle was 18 hours at 40 °C and 70 %RH followed by 6 hours at 100 °C. Source data are provided as a Source Data file.

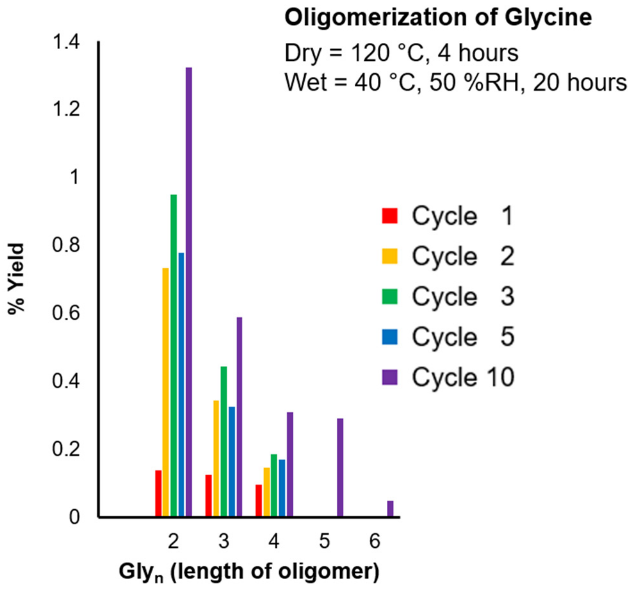

Supplementary Figure 6 | Oligomer distribution in chloride mixtures 50 %RH 120 °C. Distribution of oligomer length after cycles 1, 2, 3, 5, and 10 in the samples prepared with (K^+,Na^+)(^-Cl,^-OH). Each cycle was 20 hours at 40 °C and 50 %RH followed by 4 hours at 120 °C. Source data are provided as a Source Data file.

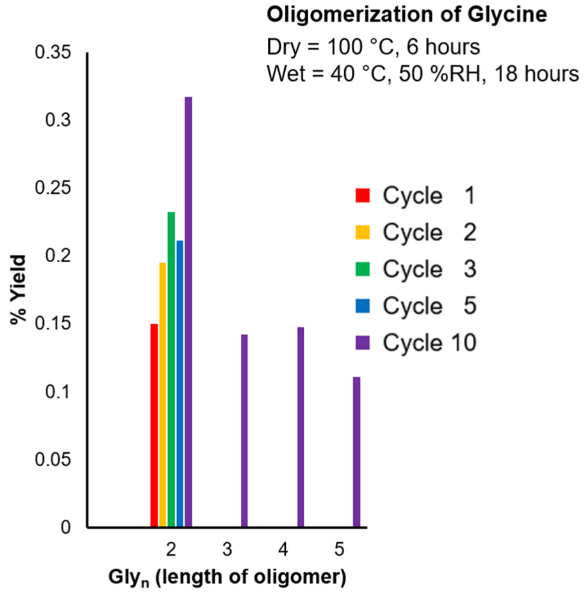

Supplementary Figure 7 | Oligomer distribution in chloride mixtures 50 %RH 100 °C. Distribution of oligomer length after cycles 1, 2, 3, 5, and 10 in the samples prepared with (K^+,Na^+)(^-Cl,^-OH). Each cycle was 18 hours at 40 °C and 50 %RH followed by 6 hours at 100 °C. Source data are provided as a Source Data file.

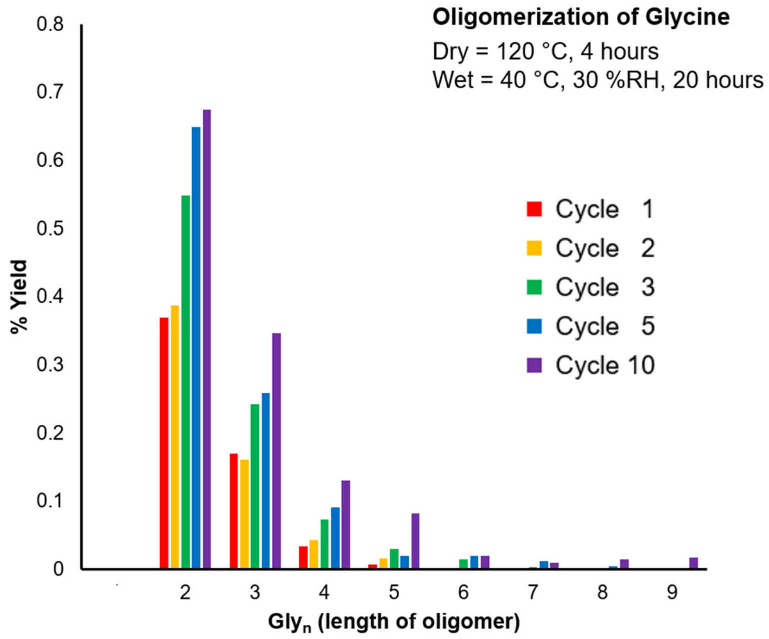

Supplementary Figure 8 | Oligomer distribution in chloride mixtures 30 %RH 120 °C. Distribution of oligomer length after cycles 1, 2, 3, 5, and 10 in the samples prepared with (K^+,Na^+)($^-$Cl,$^-$OH). Each cycle was 20 hours at 40 °C and 30 %RH followed by 4 hours at 120 °C. Source data are provided as a Source Data file.

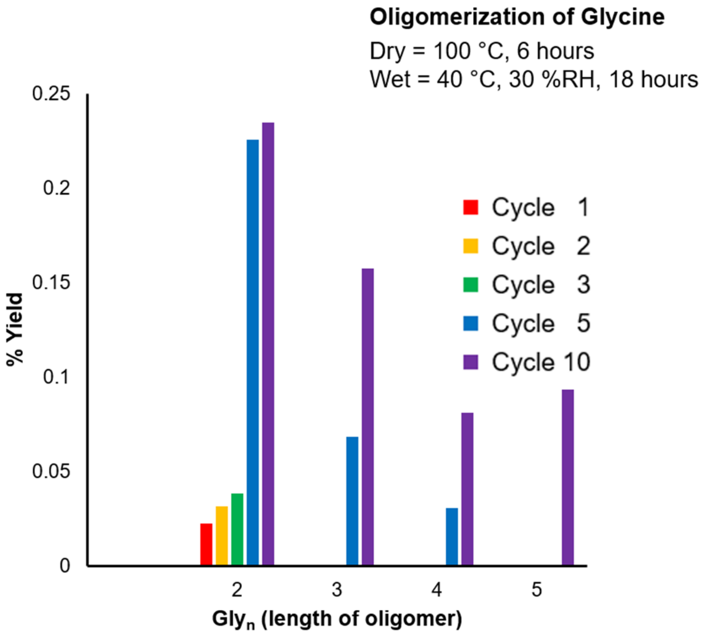

Supplementary Figure 9 | Oligomer distribution in chloride mixtures 30 %RH 100 °C. Distribution of oligomer length after cycles 1, 2, 3, 5, and 10 in the samples prepared with (K^+,Na^+)($^-$Cl,$^-$OH). Each cycle was 18 hours at 40 °C and 30 %RH followed by 6 hours at 100 °C. Source data are provided as a Source Data file.

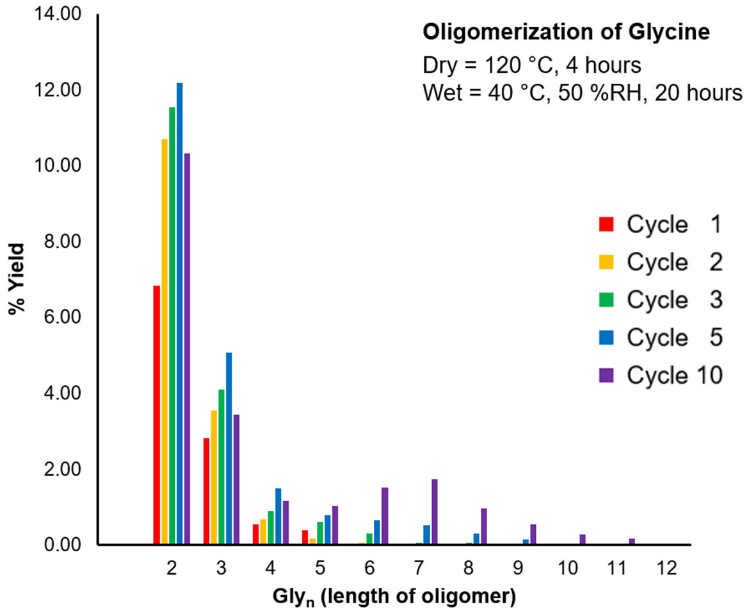

Supplementary Figure 10 | Oligomer distribution in K2HPO4 mixtures 50 %RH 120 °C. Distribution of oligomer length after cycles 1, 2, 3, 5, and 10 in the samples prepared with K_2HPO_4. Each cycle was 20 hours at 40 °C and 50 %RH followed by 4 hours at 120 °C. Source data are provided as a Source Data file.

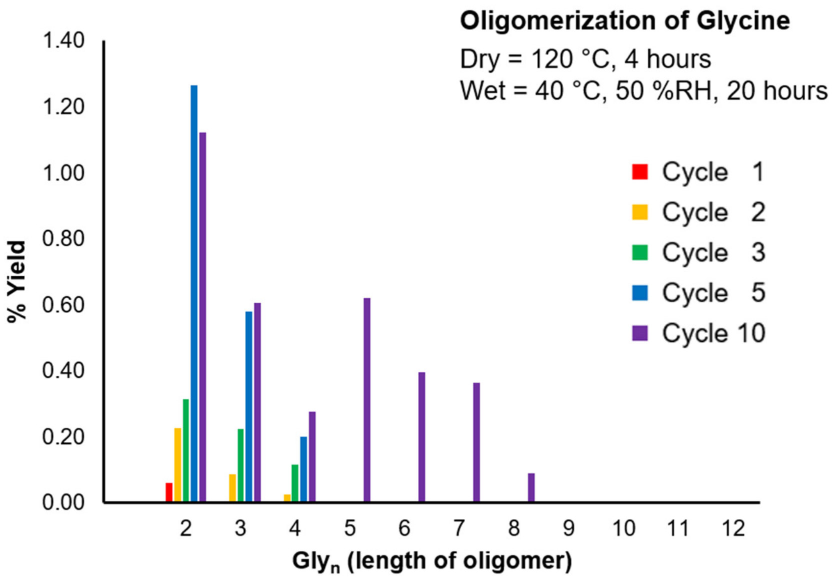

Supplementary Figure 11 | Oligomer distribution in Na_2HPO_4 mixtures 50 %RH 120 °C. Distribution of oligomer length after cycles 1, 2, 3, 5, and 10 in the samples prepared with Na_2HPO_4. Each cycle was 20 hours at 40 °C and 50 %RH followed by 4 hours at 120 °C. Source data are provided as a Source Data file.

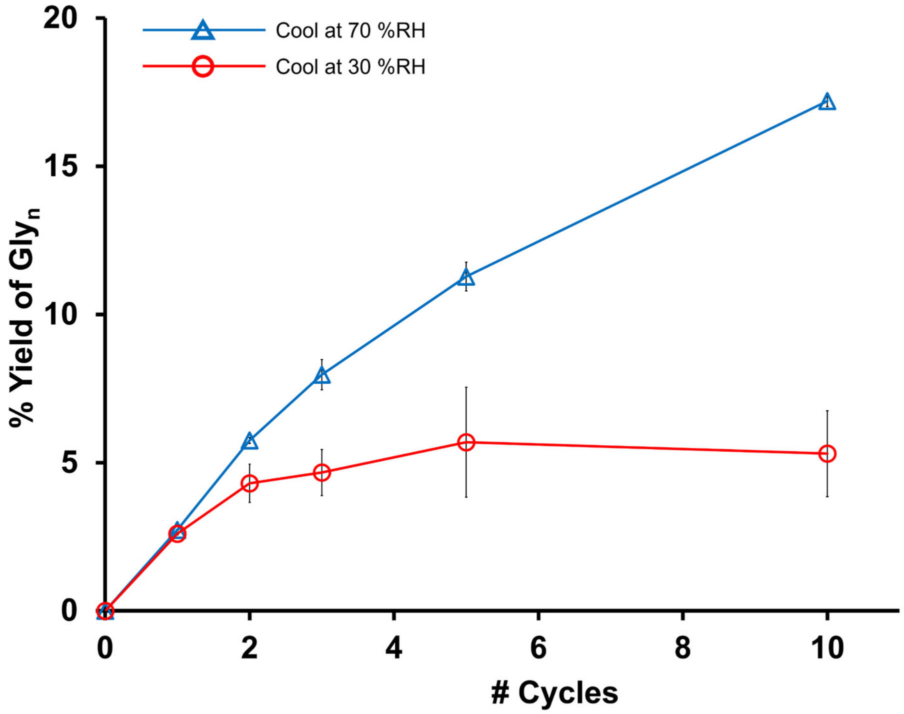

Supplementary Figure 12 | Solution-start yield comparison in chloride samples. Total yields of glycine oligomers in the presence of (K^+/Na^+)(^-Cl/^-OH) after 1, 2, 3, 5, and 10 cycles. Each cycle was 24 hours. The samples started as a stock solution of glycine and alkali salts. The first "cycle" was analyzed after 0.5 mL of the solutions were dried down at 100 °C for 6 hours. The subsequent (after the initial dry cycle) cycles included 18 hours at 40 °C and 70 %RH (blue) or 30 %RH (red) followed by 6 hours at 100 °C. The mixtures were only deliquescent at 40 °C and 70 %RH. The error bars represent 95% confidence intervals (n=6 identical experiments). Source data are provided as a Source Data file.

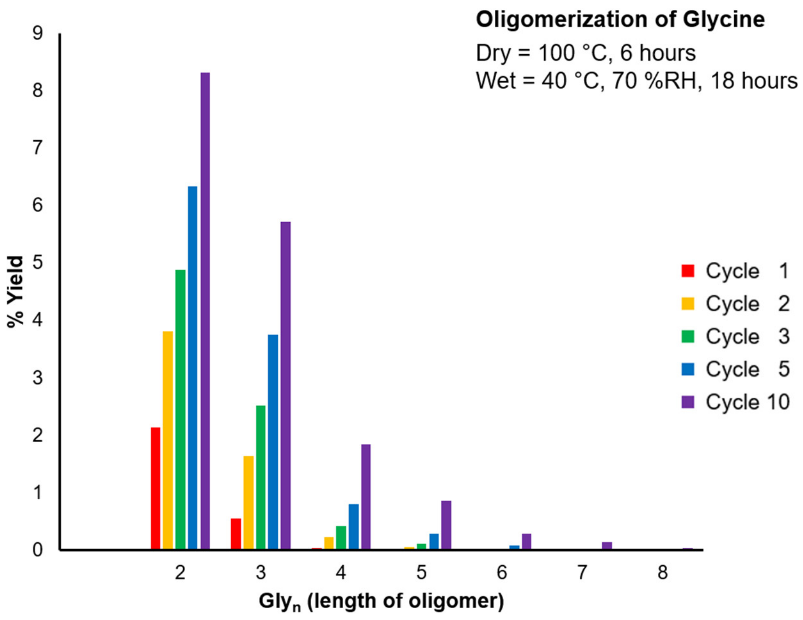

Supplementary Figure 13 | Oligomer distribution in chloride solution 70 %RH 100 °C. Distribution of oligomer length after cycles 1, 2, 3, 5, and 10 in the solution-start samples prepared with (K^+,Na^+)($^-$Cl,$^-$OH). The initial dry down of the stock solution was 6 hours at 100 °C. Each subsequent cycle was 18 hours at 40 °C and 70 %RH followed by 6 hours at 100 °C. Source data are provided as a Source Data file.

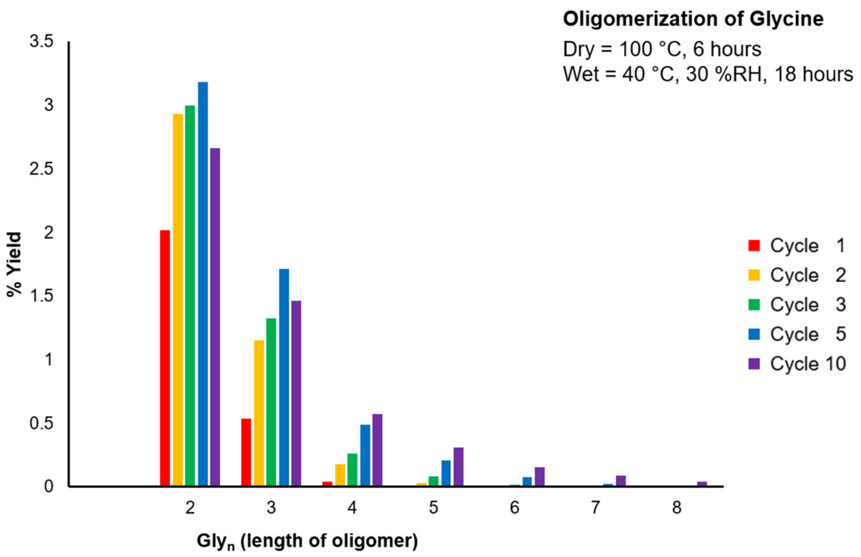

Supplementary Figure 14 | Oligomer distribution in chloride solution 30 %RH 100 °C. Distribution of oligomer length after cycles 1, 2, 3, 5, and 10 in the solution-start samples prepared with (K^+,Na^+)($^-$Cl,$^-$OH). The initial dry down of the stock solution was 6 hours at 100 °C. Each cycle was 18 hours at 40 °C and 30 %RH followed by 6 hours at 100 °C. Source data are provided as a Source Data file.

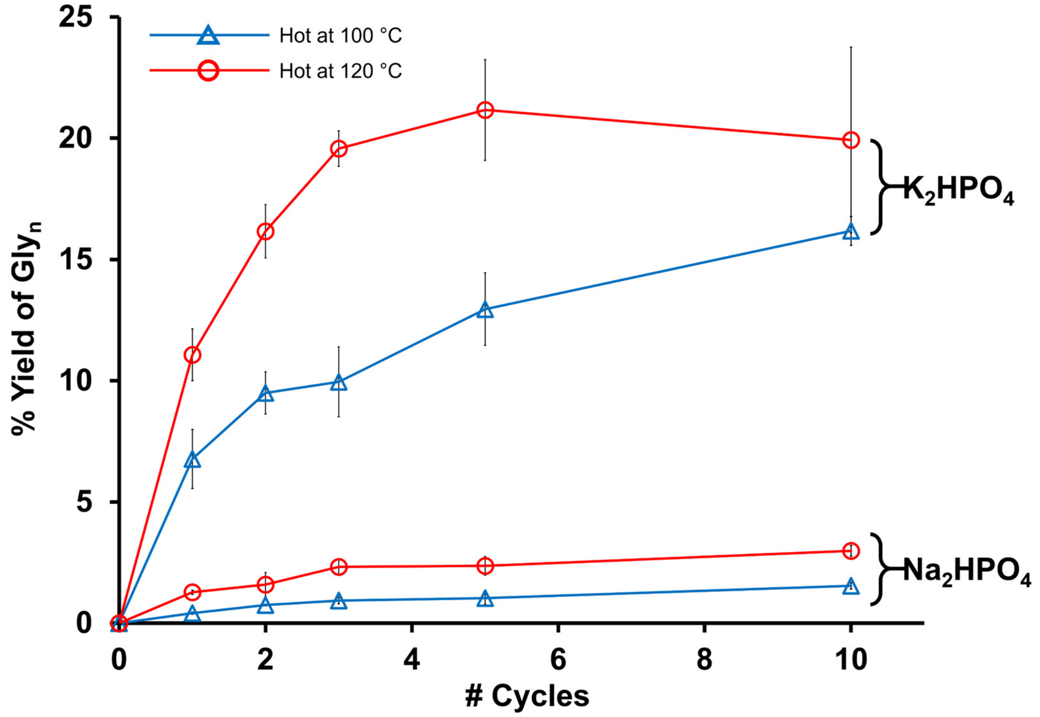

Supplementary Figure 15 | Solution-start yield comparison in dibasic phosphate samples. Total yields of glycine oligomers in the presence of K_2HPO_4 vs. Na_2HPO_4 after 1, 2, 3, 5, and 10 cycles. Each cycle was 24 hours. The samples started as homogeneous solutions of glycine and alkali salts. The first "cycle" was analyzed after 0.5 mL of the solutions were dried down at 100 °C for 6 hours. The subsequent (after the initial dry cycle) cycles included 18 hours at 40 °C and 50 %RH followed by 6 hours at 100 °C. The error bars represent 95% confidence intervals (n=6 identical experiments). Source data are provided as a Source Data file.

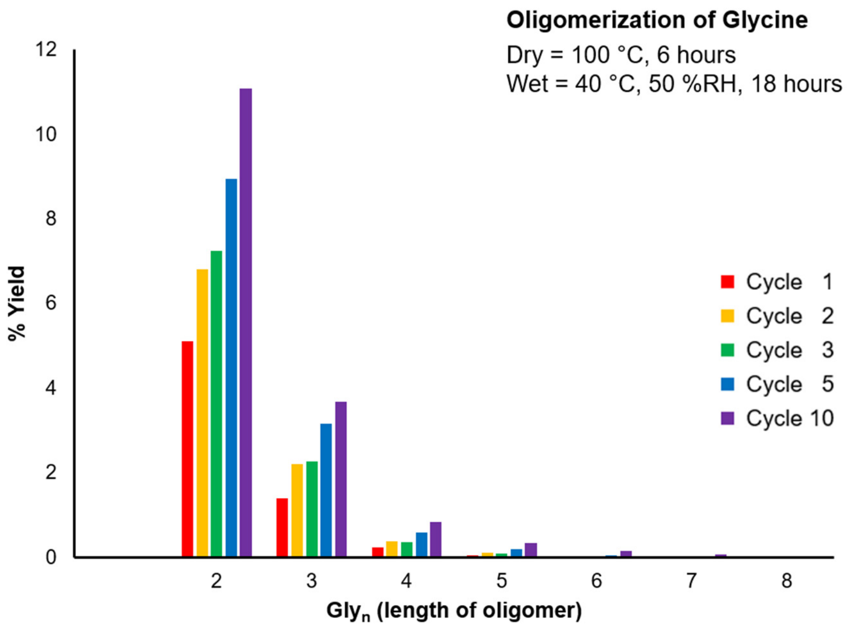

Supplementary Figure 16 | Oligomer distribution in K_2HPO_4 solution 50 %RH 100 °C. Distribution of oligomer length after cycles 1, 2, 3, 5, and 10 in the solution-start samples prepared with K_2HPO_4. The initial dry down of the stock solution was 6 hours at 100 °C. Each of the subsequent cycles was 18 hours at 40 °C and 50 %RH followed by 6 hours at 100 °C. Source data are provided as a Source Data file.

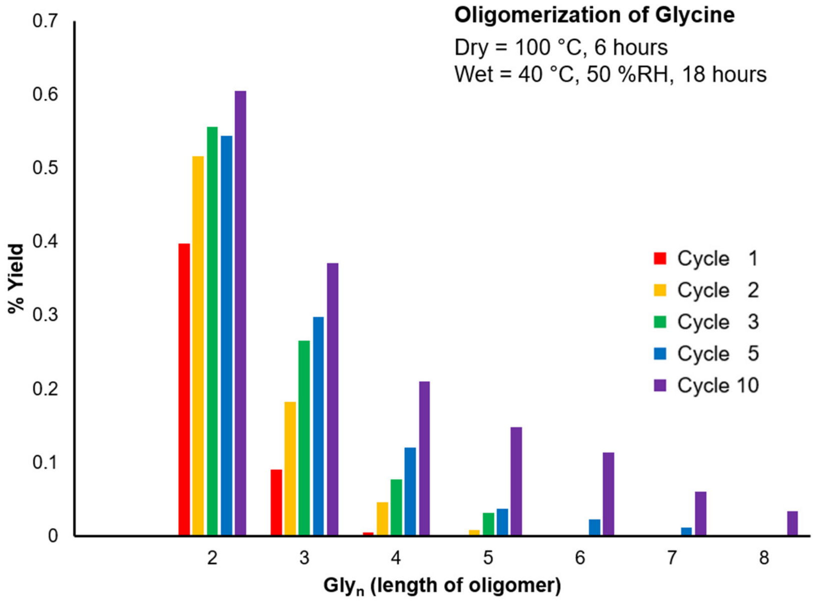

Supplementary Figure 17 | Oligomer distribution in Na_2HPO_4 solution 50 %RH 100 °C. Distribution of oligomer length after cycles 1, 2, 3, 5, and 10 in the solution-start samples prepared with Na_2HPO_4. The initial dry down of the stock solution was 6 hours at 100 °C. Each of the subsequent cycles was 18 hours at 40 °C and 50 %RH followed by 6 hours at 100 °C. Source data are provided as a Source Data file.

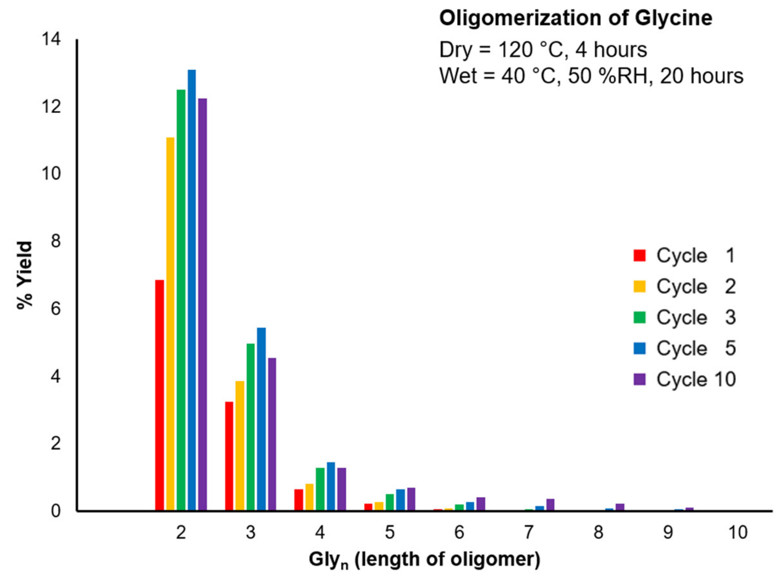

Supplementary Figure 18 | Oligomer distribution in K_2HPO_4 solution 50 %RH 120 °C. Distribution of oligomer length after cycles 1, 2, 3, 5, and 10 in the solution-start samples prepared with K_2HPO_4. The initial dry down of the stock solution was 4 hours at 120 °C. Each of the subsequent cycles was 20 hours at 40 °C and 50 %RH followed by 4 hours at 120 °C. Source data are provided as a Source Data file.

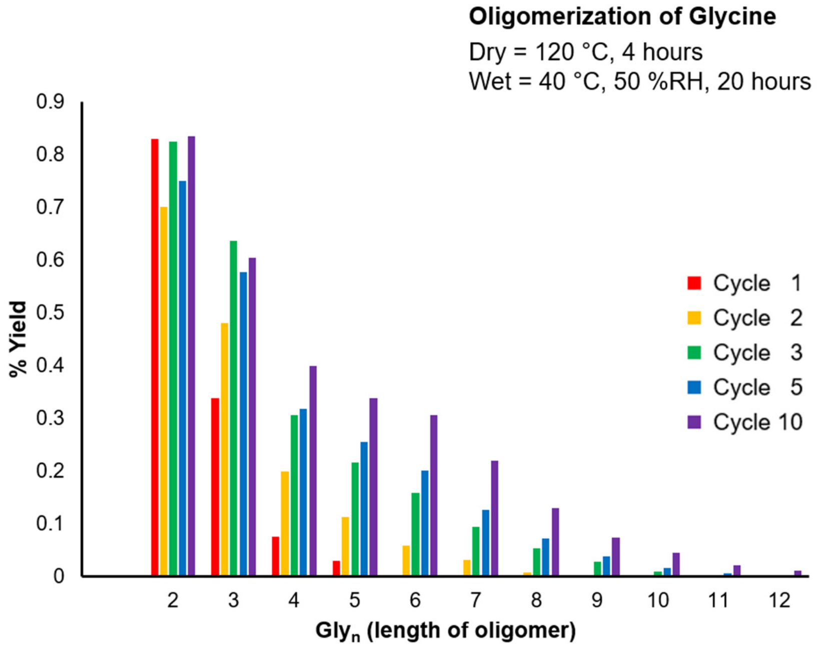

Supplementary Figure 19 | Oligomer distribution in Na_2HPO_4 solution 50 %RH 120 °C. Distribution of oligomer length after cycles 1, 2, 3, 5, and 10 in the solution-start samples prepared with Na_2HPO_4. The initial dry down of the stock solution was 4 hours at 120 °C. Each of the subsequent cycles was 20 hours at 40 °C and 50 %RH followed by 4 hours at 120 °C. Source data are provided as a Source Data file.

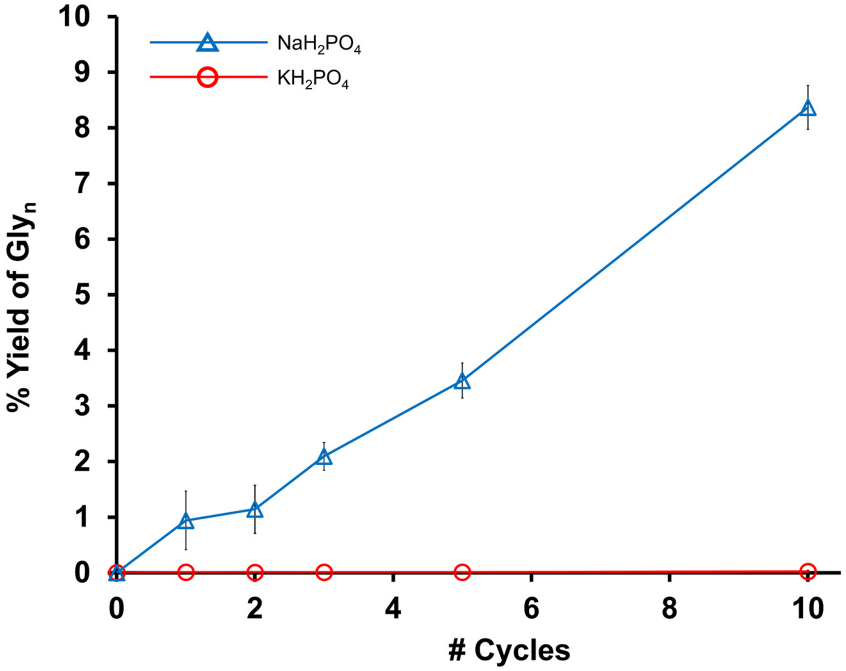

Supplementary Figure 20 | Yield comparison in monobasic phosphate samples. Total yields of glycine oligomers in the presence of KH_2PO_4 vs. NaH_2PO_4 after 1, 2, 3, 5, and 10 cycles. Each cycle was 24 hours. The samples started as a solid stock mixture of glycine and alkali salts. Each cycle included 18 hours at 40 °C and 70 %RH followed by 6 hours at 100 °C. The blue plot represents the sample conversion with KH_2PO_4 present, while the red plot represents the sample conversion with NaH_2PO_4 present. The NaH_2PO_4 mixtures were deliquescent at 40 °C and 70 %RH, while the KH_2PO_4 mixtures were not. The error bars represent 95% confidence intervals (n=3 identical experiments). Source data are provided as a Source Data file.

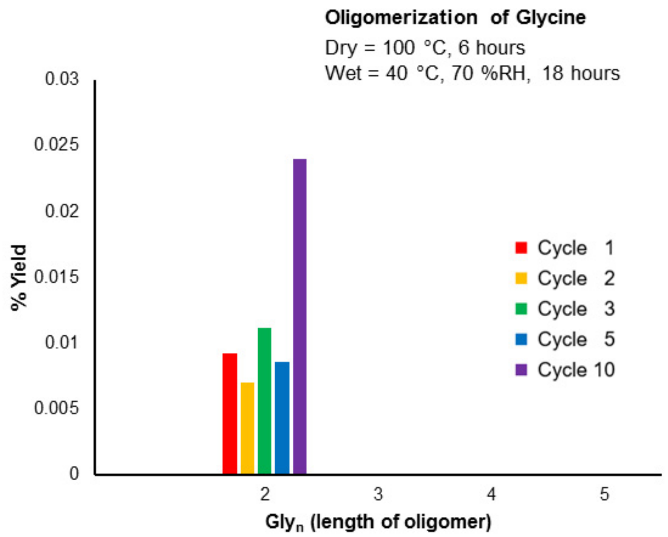

Supplementary Figure 21 | Oligomer distribution in KH_2PO_4 mixtures 70 %RH 100 °C. Distribution of oligomer length after cycles 1, 2, 3, 5, and 10 in the samples prepared with KH_2PO_4. Each cycle was 18 hours at 40 °C and 70 %RH followed by 6 hours at 100 °C. Source data are provided as a Source Data file.

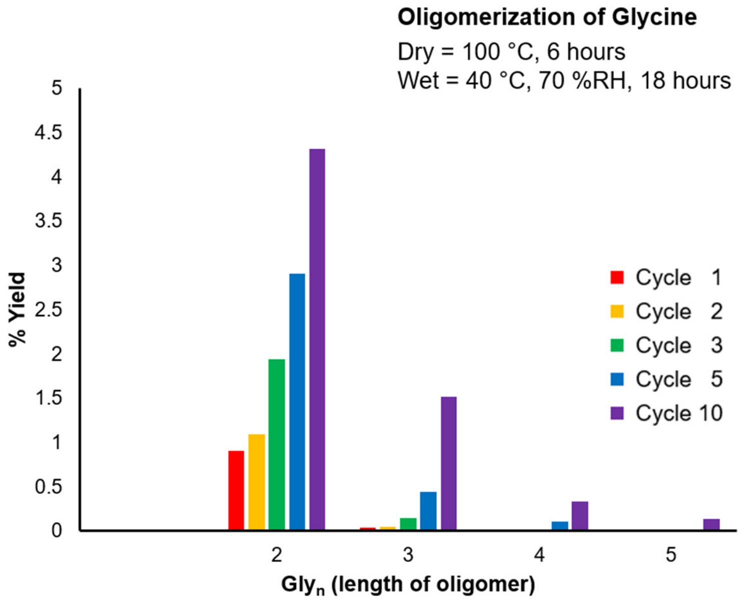

Supplementary Figure 22 | Oligomer distribution in NaH_2PO_4 mixtures 70 %RH 100 °C. Distribution of oligomer length after cycles 1, 2, 3, 5, and 10 in the samples prepared with NaH_2PO_4. Each cycle was 18 hours at 40 °C and 70 %RH followed by 6 hours at 100 °C. Source data are provided as a Source Data file.

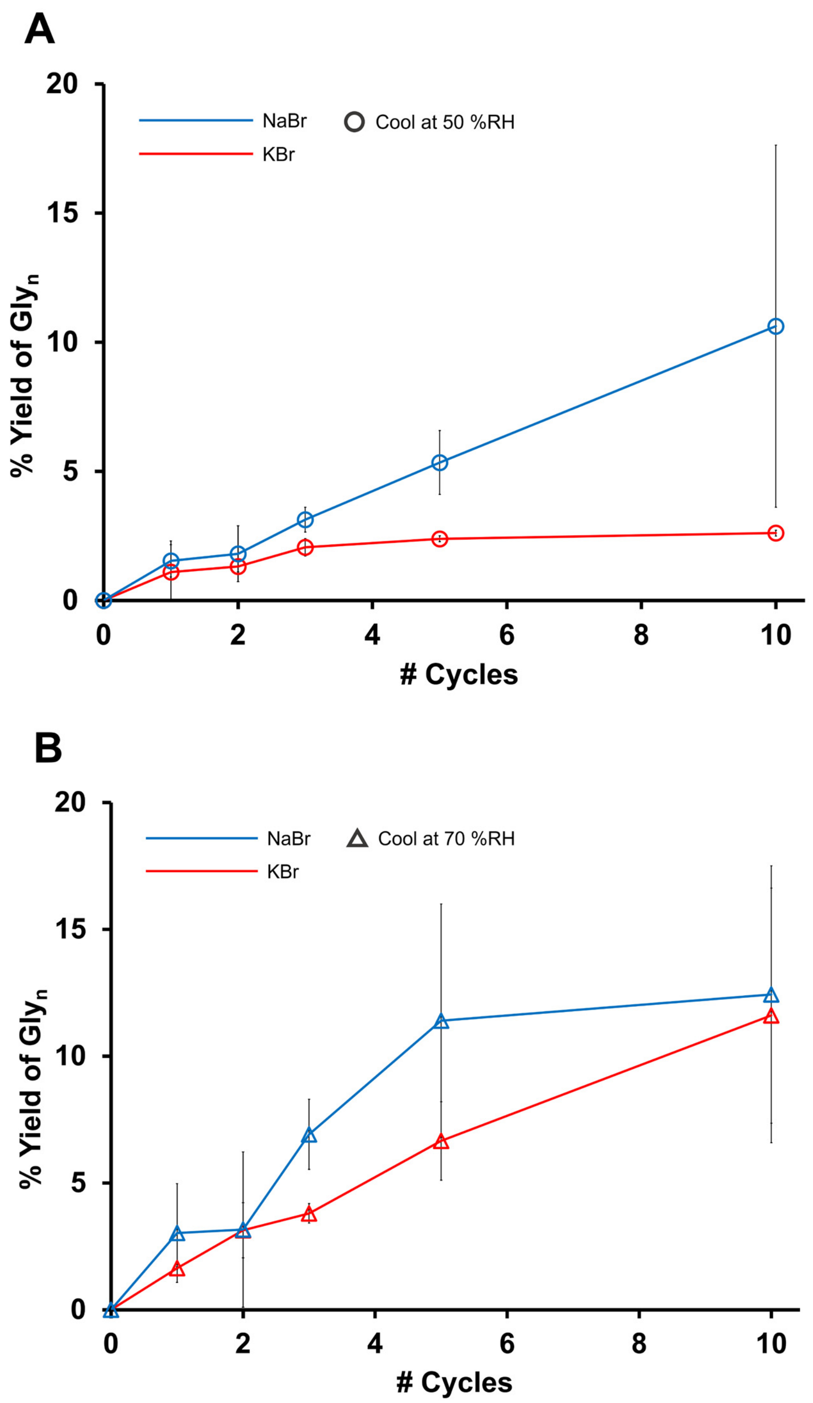

Supplementary Figure 23 | Yield comparison in bromide samples. Total yields of glycine oligomers in the presence of K(Br,OH) versus Na(Br,OH) after 1, 2, 3, 5, and 10 cycles. Each cycle was 24 hours. The samples started as a solid stock mixture of glycine and alkali salts. Each cycle was 18 hours at 40 °C and either (a) 50 %RH or (b) 70 %RH, followed by 6 hours at 100 °C. The blue plot represents the sample conversion with NaBr present, while the red plot represents the sample conversion with KBr present. Both mixtures were deliquescent at 40 °C and 70 %RH. The error bars represent 95% confidence intervals (n=3 identical experiments). Source data are provided as a Source Data file.

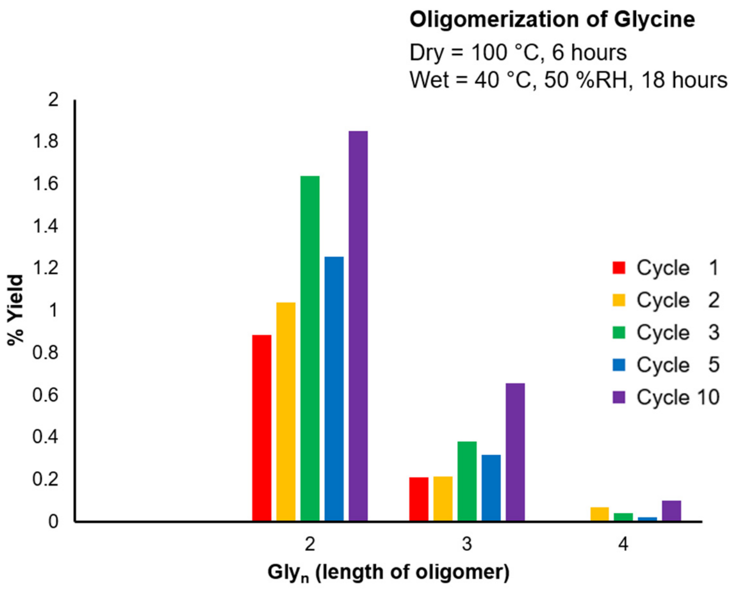

Supplementary Figure 24 | Oligomer distribution in KBr mixtures 50 %RH 100 °C. Distribution of oligomer length after cycles 1, 2, 3, 5, and 10 in the samples prepared with KBr. Each cycle was 18 hours at 40 °C and 50 %RH followed by 6 hours at 100 °C. Source data are provided as a Source Data file.

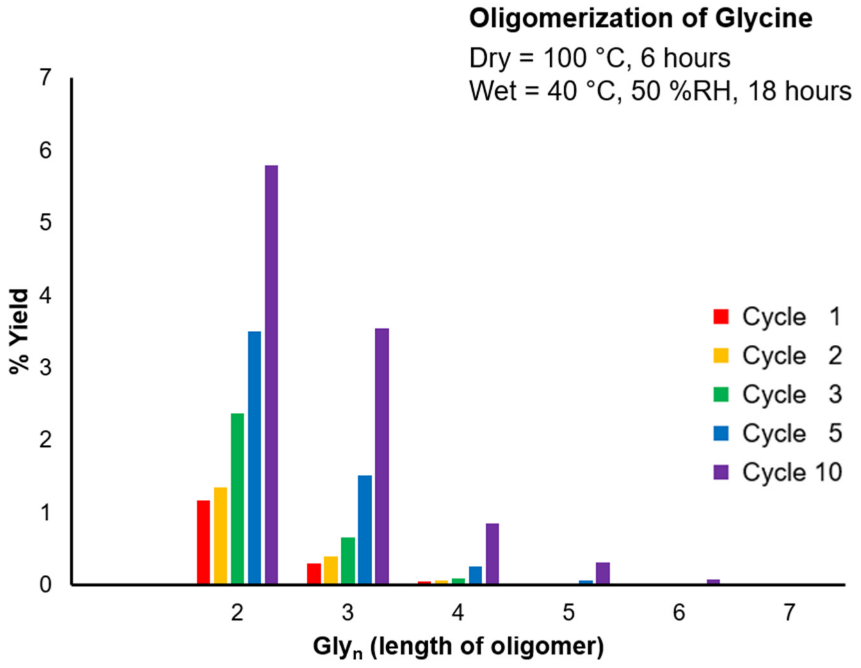

Supplementary Figure 25 | Oligomer distribution in NaBr mixtures 50 %RH 100 °C. Distribution of oligomer length after cycles 1, 2, 3, 5, and 10 in the samples prepared with NaBr. Each cycle was 18 hours at 40 °C and 50 %RH followed by 6 hours at 100 °C. Source data are provided as a Source Data file.

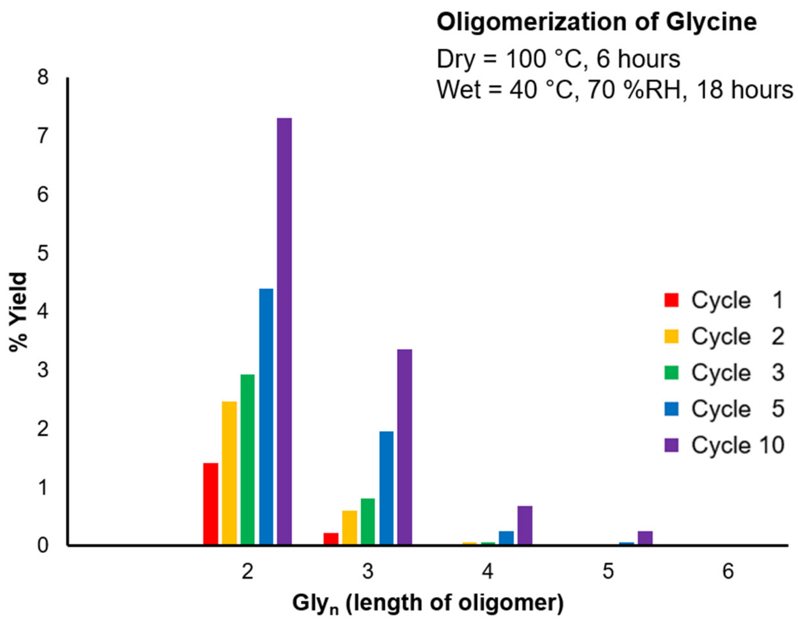

Supplementary Figure 26 | Oligomer distribution in KBr mixtures 70 %RH 100 °C. Distribution of oligomer length after cycles 1, 2, 3, 5, and 10 in the samples prepared with KBr. Each cycle was 18 hours at 40 °C and 70 %RH followed by 6 hours at 100 °C. Source data are provided as a Source Data file.

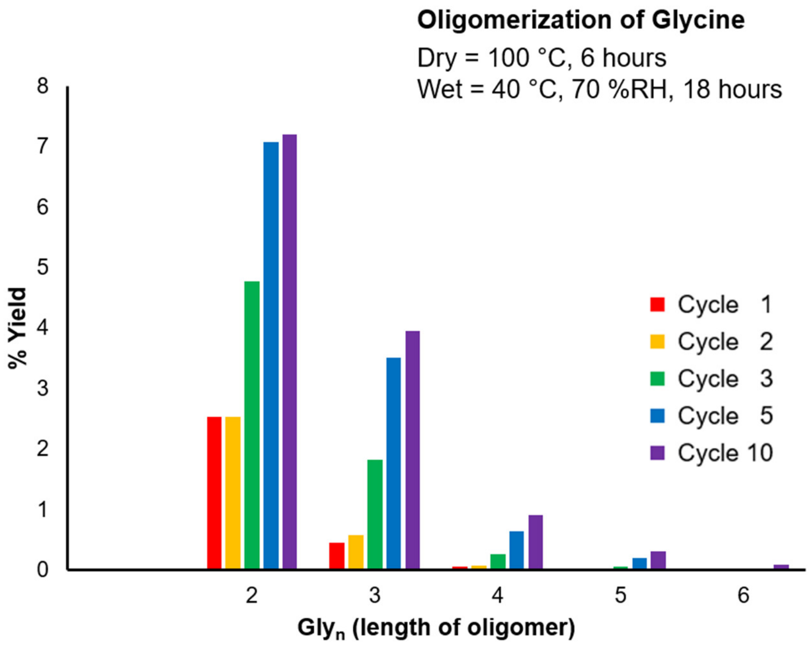

Supplementary Figure 27 | Oligomer distribution in KBr mixtures 70 %RH 100 °C. Distribution of oligomer length after cycles 1, 2, 3, 5, and 10 in the samples prepared with NaBr. Each cycle was 18 hours at 40 °C and 70 %RH followed by 6 hours at 100 °C. Source data are provided as a Source Data file.

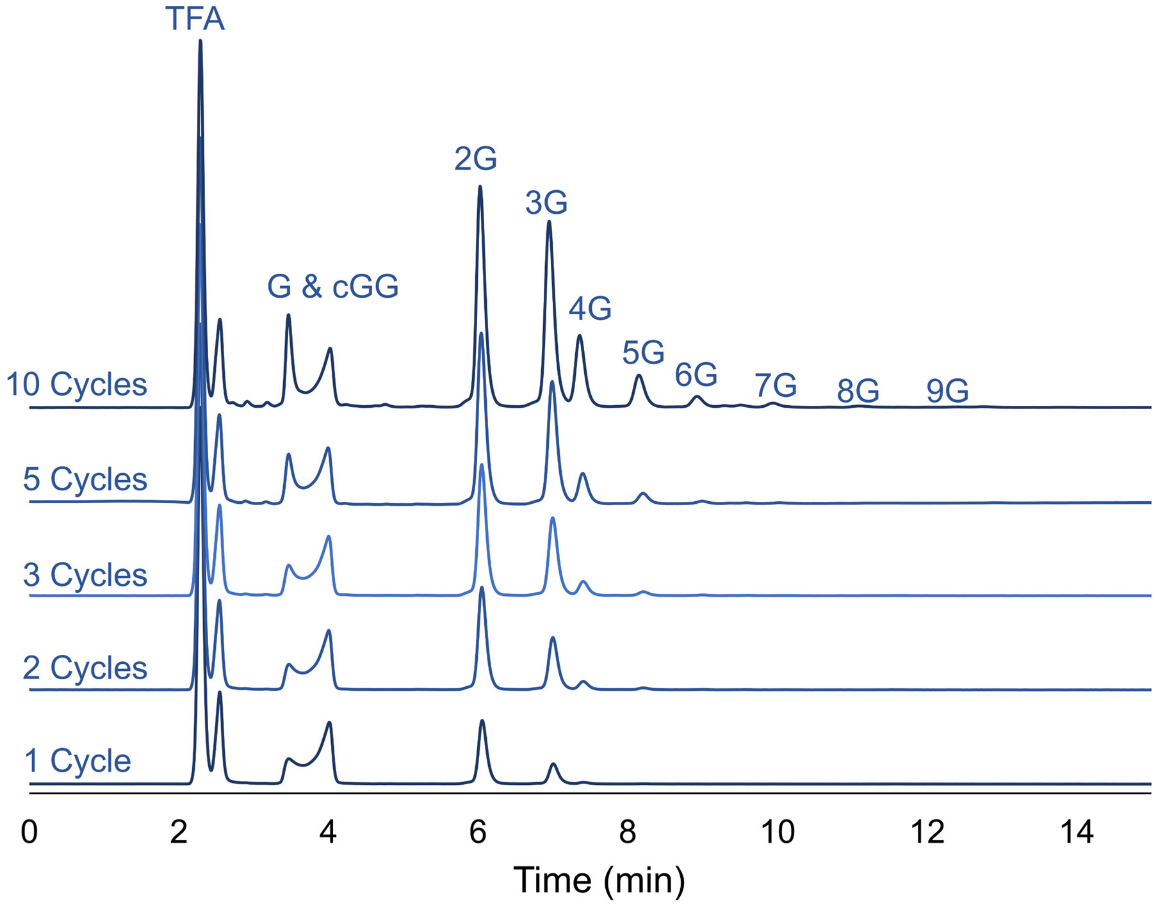

Supplementary Figure 28 | Example chromatograms of glycine oligomerization. Chromatograms were obtained after cycles 1, 2, 3, 5, and 10 from samples prepared with $(Na^+,K^+)(^-Cl,OH^-)$. Each cycle was 18 hours at 40 °C and 70 %RH followed by 6 hours at 100 °C. Source data are provided as a Source Data file.

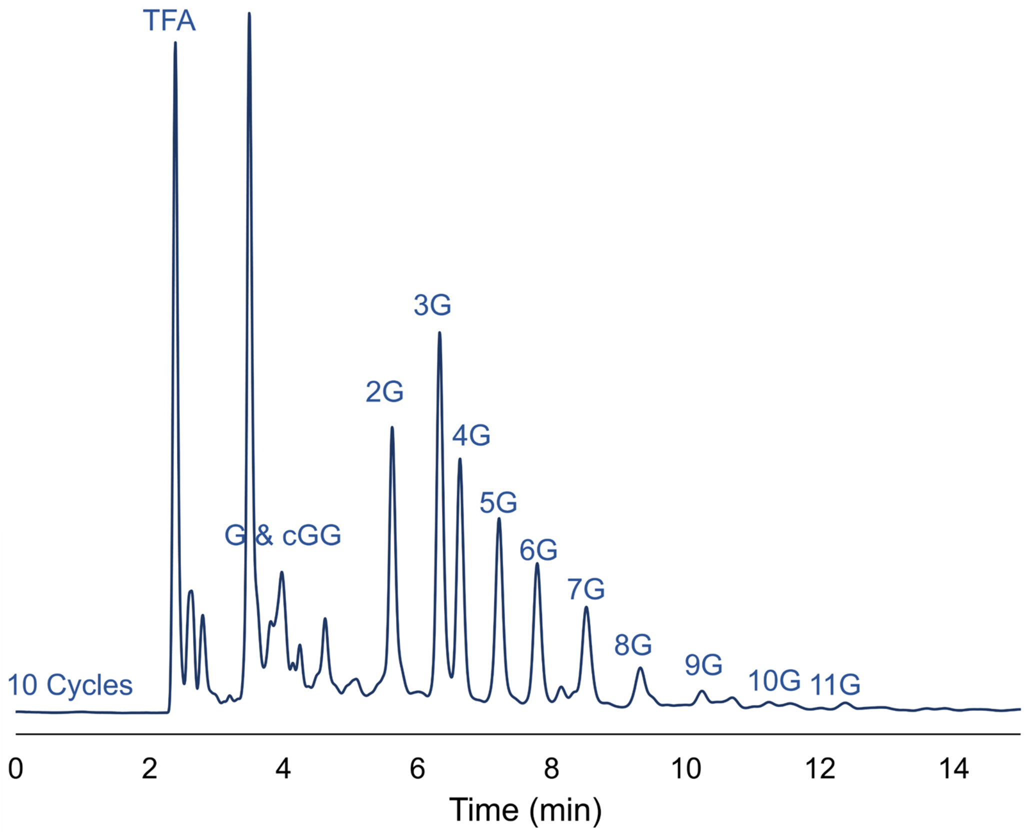

Supplementary Figure 29 | Chromatogram of the MALDI sample. Chromatogram of glycine oligomerization reaction after cycle 15 in the sample prepared with (Na^+,K^+)($^-$Cl, $^-$OH). Each cycle was 20 hours at 40 °C and 70 %RH followed by 4 hours at 120 °C. Source data are provided as a Source Data file.

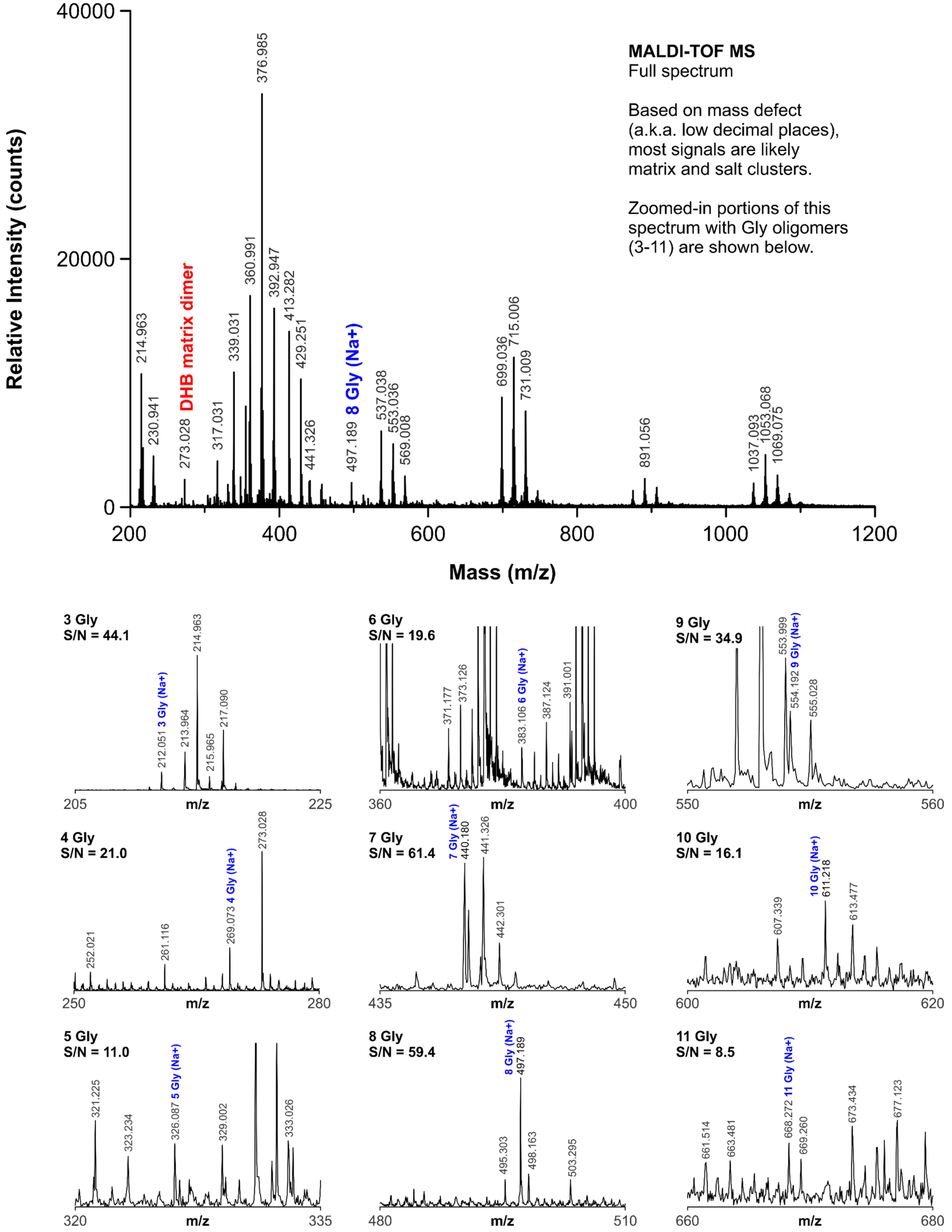

Supplementary Figure 30 | MALDI–TOF data.

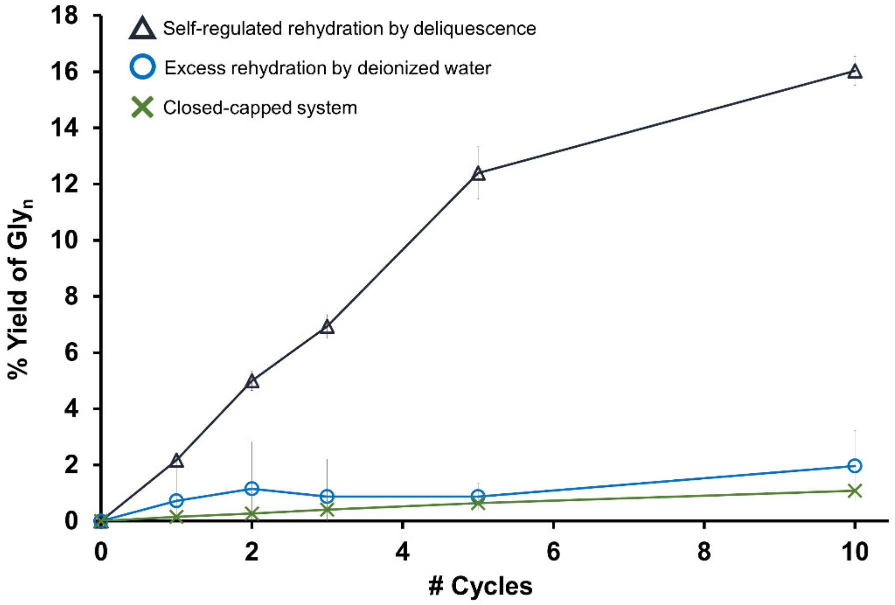

Supplementary Figure 31 | Yield comparison of various hydration models. Yields of glycine oligomers (excluding DKP) in the presence of (K^+,Na^+)(^-Cl, ^-OH) after 1, 2, 3, 5, and 10 cycles. Each cycle was 24 hours. For the samples marked with triangles, one cycle included 18 hours at 40 °C and 70 %RH, followed by 6 hours at 100 °C. The samples marked with circles were exposed to the same environmental cycles, but 20 mL of water was added to the mixture before each drying period. This addition simulated heavy rain and overhydration of the sample to verify a shortcoming of the standard model for wet–dry cycling that is obviated by self-regulated, limited rehydration through deliquescence. The samples marked with crosses represent the closed-capped system, which tested the propensity for glycine oligomerization in samples not permitted to evaporate during the hot phase of each cycle. The error bars represent 95% confidence intervals (n=3 identical experiments). Source data are provided as a Source Data file.

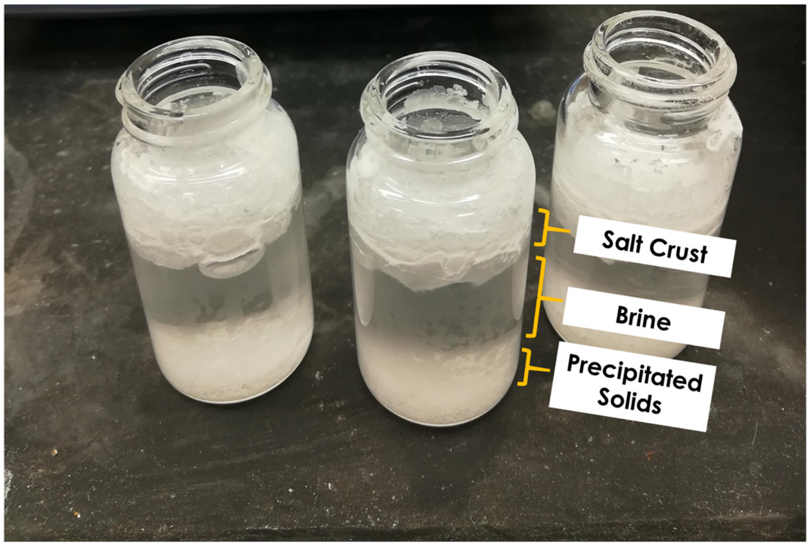

Supplementary Figure 32 | Brine overhydration samples. Photograph of the reaction vials in the overhydration with brine experiment described in Supplementary Methods. During evaporation, a crust of salt sealed the liquid reaction mixture, preventing evaporation to dryness during the hot phase of each cycle.

Supplementary Tables

Supplementary Table 1 | Summary of the glycine wet–dry cycling with various salts. Results of glycine oligomerization by wet–dry cycling when starting from a mixture of solids. The salts present, conditions of each cycle, and whether the mixture was deliquescent in the wet conditions are indicated in the first four columns. The total yield of all Gly_n oligomers with $n \geq 2$ (not including DKP) for each experiment is reported as a 95% confidence interval based (n=3 identical experiments). The longest Gly_n oligomer observed by IP–HPLC is also noted. Each entry is cross-referenced against the figures that track the progress of the reactions over the ten cycles of the experiment. Source data are provided as a Source Data file.

Salt(s)	Dry Phase	Wet Phase (all 40 °C)	Deliquescent?	Yield After 10 Cycles (%)	Longest Gly_n Observed	Graphical Data
$(K^+,Na^+)(Cl^-,OH^-)$	4 h @ 120 °C	20 h @ 70 %RH	Y	17.3 ± 0.4	13	Fig. 2, Supplementary Figs. 4–9
	6 h @ 100 °C	18 h @ 70 %RH	Y	16.0 ± 0.6	13	
	4 h @ 120 °C	20 h @ 50 %RH	N	2.6 ± 0.2	6	
	6 h @ 100 °C	18 h @ 50 %RH	N	0.7 ± 1.5	5	
	4 h @ 120 °C	20 h @ 30 %RH	N	1.3 ± 0.2	9	
	6 h @ 100 °C	18 h @ 30 %RH	N	0.6 ± 0.1	5	
K_2HPO_4	4 h @ 120 °C	20 h @ 50 %RH	Y	21.2 ± 1.9	11	Fig. 4, Supplementary Figs. 10, 11
Na_2HPO_4	4 h @ 120 °C	20 h @ 50 %RH	N	3.5 ± 0.5	8	
KH_2PO_4	6 h @ 100 °C	18 h @ 70 %RH	N	0.0 ± 0.0	0	Supplementary Figs. 21, 22
NaH_2PO_4	6 h @ 100 °C	18 h @ 70 %RH	Y	6.3 ± 0.4	5	
$(K^+)(Br^-,OH^-)$	6 h @ 100 °C	18 h @ 70 %RH	Y	11.6 ± 5.0	6	Supplementary Figs. 23, 24, 26, 27
	6 h @ 100 °C	18 h @ 50 %RH	N†	2.6 ± 0.1	4	
$(Na^+)(Br^-,OH^-)$	6 h @ 100 °C	18 h @ 70 %RH	Y	12.4 ± 5.1	6	
	6 h @ 100 °C	18 h @ 50 %RH	Y	10.5 ± 7.0	7	

† The mixture of KBr & KOH was not deliquescent at 50 %RH, but the solid did acquire some moisture and form clumps.

Supplementary Table 2 | Summary of the glycine solution-start wet–dry cycling. Results of glycine oligomerization by wet–dry cycling when starting from homogeneous solutions. The salts present, conditions of each cycle, and whether the mixture was deliquescent in the wet conditions are indicated in the first four columns. The total yield of all Gly_n oligomers for $n \geq 2$ (not including DKP) for each experiment is reported as a 95% confidence interval (n=3 identical experiments). The longest Gly_n oligomer observed by IP–HPLC is also noted. Each entry is cross-referenced against the figures that track the progress of the reactions over the ten cycles of the experiment. Source data are provided as a Source Data file.

Salt(s)	Dry Phase	Wet Phase (all 40 °C)	Deliquescent?	Yield After 10 Cycles (%)	Longest Gly_n Observed	Graphical Data
$(K^+,Na^+)(Cl^-,OH^-)$	6 h @ 100 °C	18 h @ 70 %RH	Y	17.2 ± 0.6	8	Supplementary Figs. 12–14
	6 h @ 100 °C	18 h @ 30 %RH	N	5.3 ± 1.5	9	
K_2HPO_4	4 h @ 120 °C	20 h @ 50 %RH	Y	19.9 ± 3.8	10	Supplementary Figs. 15, 16, 18
	6 h @ 100 °C	18 h @ 50 %RH	Y	16.2 ± 2.8	8	
Na_2HPO_4	4 h @ 120 °C	20 h @ 50 %RH	N	3.0 ± 0.2	12	Supplementary Figs. 15, 17, 19
	6 h @ 100 °C	18 h @ 50 %RH	N	1.5 ± 0.5	8	

Supplementary Table 3 | Summary of the data shown in Supplementary Figure 1. The salts which are deliquescent are denoted with a plus sign (+) in a blue cell while those that were not deliquescent are denoted by a minus sign (−) in a white cell.

	30 %RH		50 %RH		70 %RH	
	Na^+	K^+	Na^+	K^+	Na^+	K^+
F^-	−	+	−	+	−	+
Cl^-	−	−	−	−	−	−
Br^-	−	−	−	−	+	−
I^-	+	−	+	−	+	+
CO_3^{2-}	−	−	−	+	−	+
HCO_3^-	−	−	−	−	−	−
$CH_3CO_2^-$	−	+	−	+	+	+
HCO_2^-	−	+	−	+	+	+
$H_2PO_4^-$	−	−	−	−	+	−
HPO_4^{2-}	−	+	−	+	−	+
PO_4^{3-}	−	+	−	+	−	+
$P_2O_7^{4-}$	−	+	−	+	−	+

Supplementary Table 4 | Summary of the data shown in Supplementary Figure 4. Distribution of oligomer length after cycles 1, 2, 3, 5, and 10 in the samples prepared with (Na,K)(Cl,OH). Each cycle was 20 hours at 40 °C and 70 %RH followed by 4 hours at 120 °C. Oligomers without entries for a particular cycle were not produced in a quantity greater than their limit of quantitation by our method of analysis. Source data are provided as a Source Data file.

	**Average Yield After Each Cycle (%)**				
Gly_n	Cycle 1	Cycle 2	Cycle 3	Cycle 5	Cycle 10
2	5.30	6.99	7.72	5.86	4.77
3	3.87	5.86	7.04	5.61	4.07
4	0.90	1.80	2.68	2.96	2.52
5	0.26	0.81	1.38	2.12	1.94
6	0.07	0.28	0.55	1.29	1.35
7	0.02	0.13	0.29	1.09	1.23
8	0.01	0.07	0.25	0.61	0.71
9		0.01	0.13	0.35	0.31
10		0.01	0.08	0.18	0.17
11				0.12	0.15
12				0.07	0.06
13				0.07	0.05
Total	10.43	15.96	20.12	20.33	17.33

Supplementary Table 5 | Summary of the data shown in Supplementary Figure 5. Distribution of oligomer length after cycles 1, 2, 3, 5, and 10 in the samples prepared with (Na,K)(Cl,OH). Each cycle was 18 hours at 40 °C and 70 %RH followed by 6 hours at 100 °C. Oligomers without entries for a particular cycle were not produced in a quantity greater than their limit of quantitation by our method of analysis. Source data are provided as a Source Data file.

	**Average Yield After Each Cycle (%)**				
Gly_n	Cycle 1	Cycle 2	Cycle 3	Cycle 5	Cycle 10
2	1.77	3.48	4.43	5.45	5.96
3	0.35	1.32	2.09	4.43	4.67
4	0.02	0.16	0.33	1.35	2.21
5		0.03	0.08	0.68	1.36
6			0.02	0.25	0.65
7			0.01	0.11	0.44
8				0.08	0.32
9				0.03	0.15
10					0.07
11					0.07
12					0.05
13					0.02
Total	2.14	4.99	6.96	12.38	15.97

Supplementary Table 6 | Summary of the data shown in Supplementary Figure 6. Distribution of oligomer length after cycles 1, 2, 3, 5, and 10 in the samples prepared with (Na,K)(Cl,OH). Each cycle was 20 hours at 40 °C and 50 %RH followed by 4 hours at 120 °C. Oligomers without entries for a particular cycle were not produced in a quantity greater than their limit of quantitation by our method of analysis. Source data are provided as a Source Data file.

	**Average Yield After Each Cycle (%)**				
Gly_n	Cycle 1	Cycle 2	Cycle 3	Cycle 5	Cycle 10
2	0.14	0.73	0.95	0.78	1.32
3	0.12	0.34	0.44	0.33	0.59
4	0.10	0.14	0.18	0.17	0.31
5					0.29
6					0.05
Total	0.36	1.22	1.58	1.27	2.56

Supplementary Table 7 | Summary of the data shown in Supplementary Figure 7. Distribution Distribution of oligomer length after cycles 1, 2, 3, 5, and 10 in the samples prepared with (Na,K)(Cl,OH). Each cycle was 18 hours at 40 °C and 50 %RH followed by 6 hours at 100 °C. Oligomers without entries for a particular cycle were not produced in a quantity greater than their limit of quantitation by our method of analysis. Source data are provided as a Source Data file.

	**Average Yield After Each Cycle (%)**				
Gly_n	Cycle 1	Cycle 2	Cycle 3	Cycle 5	Cycle 10
2	0.15	0.20	0.23	0.21	0.32
3					0.14
4					0.15
5					0.11
Total	0.15	0.20	0.23	0.21	0.72

Supplementary Table 8 | Summary of the data shown in Supplementary Figure 8. Distribution of oligomer length after cycles 1, 2, 3, 5, and 10 in the samples prepared with (Na,K)(Cl,OH). Each cycle was 20 hours at 40 °C and 30 %RH followed by 4 hours at 120 °C. Oligomers without entries for a particular cycle were not produced in a quantity greater than their limit of quantitation by our method of analysis. Source data are provided as a Source Data file.

	**Average Yield After Each Cycle (%)**				
Gly_n	Cycle 1	Cycle 2	Cycle 3	Cycle 5	Cycle 10
2	0.37	0.39	0.55	0.65	0.67
3	0.17	0.16	0.24	0.26	0.35
4	0.03	0.04	0.07	0.09	0.13
5	0.01	0.02	0.03	0.02	0.08
6			0.01	0.02	0.02
7				0.01	0.01
8					0.01
9					0.02
Total	0.58	0.61	0.91	1.05	1.29

Supplementary Table 9 | Summary of the data shown in Supplementary Figure 9. Distribution of oligomer length after cycles 1, 2, 3, 5, and 10 in the samples prepared with (Na,K)(Cl,OH). Each cycle was 18 hours at 40 °C and 30 %RH followed by 6 hours at 100 °C. Oligomers without entries for a particular cycle were not produced in a quantity greater than their limit of quantitation by our method of analysis. Source data are provided as a Source Data file.

	**Average Yield After Each Cycle (%)**				
Gly_n	Cycle 1	Cycle 2	Cycle 3	Cycle 5	Cycle 10
2	0.02	0.03	0.04	0.23	0.23
3				0.07	0.16
4				0.03	0.08
5					0.09
Total	0.02	0.03	0.04	0.32	0.57

Supplementary Table 10 | Summary of the data shown in Supplementary Figure 10. Distribution of oligomer length after cycles 1, 2, 3, 5, and 10 in the samples prepared with K_2HPO_4. Each cycle was 20 hours at 40 °C and 50 %RH followed by 4 hours at 120 °C. Oligomers without entries for a particular cycle were not produced in a quantity greater than their limit of quantitation by our method of analysis. Source data are provided as a Source Data file.

	**Average Yield After Each Cycle (%)**				
Gly_n	Cycle 1	Cycle 2	Cycle 3	Cycle 5	Cycle 10
2	6.84	10.7	11.5	12.1	10.3
3	2.82	3.55	4.11	5.07	3.44
4	0.56	0.68	0.89	1.49	1.17
5	0.39	0.18	0.61	0.79	1.04
6	0.04	0.07	0.30	0.65	1.52
7			0.07	0.53	1.74
8			0.07	0.30	0.97
9				0.16	0.56
10					0.29
11					0.18
Total	10.63	15.19	17.59	21.18	21.23

Supplementary Table 11 | Summary of the data shown in Supplementary Figure 11. Distribution of oligomer length after cycles 1, 2, 3, 5, and 10 in the samples prepared with Na_2HPO_4. Each cycle was 20 hours at 40 °C and 50 %RH followed by 4 hours at 120 °C. Oligomers without entries for a particular cycle were not produced in a quantity greater than their limit of quantitation by our method of analysis. Source data are provided as a Source Data file.

	**Average Yield After Each Cycle (%)**				
Gly_n	Cycle 1	Cycle 2	Cycle 3	Cycle 5	Cycle 10
2	0.06	0.23	0.31	1.26	1.12
3		0.09	0.22	0.58	0.61
4		0.02	0.12	0.20	0.28
5					0.62
6					0.40
7					0.37
8					0.09
Total	0.06	0.34	0.65	2.04	3.47

Supplementary Table 12 | Summary of the data shown in Supplementary Figure 13. Distribution of oligomer length after cycles 1, 2, 3, 5, and 10 in the solution-start samples prepared with (K^+,Na^+)(^-Cl,^-OH). The initial dry down of the stock solution was 6 hours at 100 °C. Each subsequent cycle was 18 hours at 40 °C and 70 %RH followed by 6 hours at 100 °C. Oligomers without entries for a particular cycle were not produced in a quantity greater than their limit of quantitation by our method of analysis. Source data are provided as a Source Data file.

	Average Yield After Each Cycle (%)				
Gly_n	Cycle 1	Cycle 2	Cycle 3	Cycle 5	Cycle 10
2	2.13	3.81	4.88	6.33	8.31
3	0.55	1.64	2.52	3.75	5.71
4	0.04	0.24	0.42	0.81	1.85
5		0.06	0.12	0.29	0.86
6		0.01	0.02	0.08	0.29
7				0.02	0.14
8					0.03
Total	2.73	5.75	7.97	11.28	17.20

Supplementary Table 13 | Summary of the data shown in Supplementary Figure 14. Distribution Distribution of oligomer length after cycles 1, 2, 3, 5, and 10 in the solution-start samples prepared with (K^+,Na^+)(^-Cl,^-OH). The initial dry down of the stock solution was 6 hours at 100 °C. Each subsequent cycle was 18 hours at 40 °C and 30 %RH followed by 6 hours at 100 °C. Oligomers without entries for a particular cycle were not produced in a quantity greater than their limit of quantitation by our method of analysis. Source data are provided as a Source Data file.

	Average Yield After Each Cycle (%)				
Gly_n	Cycle 1	Cycle 2	Cycle 3	Cycle 5	Cycle 10
2	2.02	2.93	3.00	3.18	2.66
3	0.54	1.15	1.32	1.71	1.46
4	0.04	0.18	0.26	0.49	0.57
5	0.01	0.03	0.08	0.21	0.31
6		0.01	0.02	0.08	0.15
7				0.03	0.09
8					0.04
9					0.01
Total	2.60	4.30	4.69	5.69	5.30

Supplementary Table 14 | Summary of the data shown in Supplementary Figure 16. Distribution of oligomer length after cycles 1, 2, 3, 5, and 10 in the solution-start samples prepared with K_2HPO_4. The initial dry down of the stock solution was 6 hours at 100 °C. Each of the subsequent cycles was 18 hours at 40 °C and 50 %RH followed by 6 hours at 100 °C. Oligomers without entries for a particular cycle were not produced in a quantity greater than their limit of quantitation by our method of analysis. Source data are provided as a Source Data file.

	**Average Yield After Each Cycle (%)**				
Gly_n	Cycle 1	Cycle 2	Cycle 3	Cycle 5	Cycle 10
2	5.10	6.81	7.25	8.95	11.08
3	1.39	2.20	2.25	3.16	3.68
4	0.23	0.38	0.36	0.59	0.84
5	0.05	0.11	0.09	0.18	0.34
6			0.02	0.05	0.15
7				0.02	0.06
8					0.01
Total	6.77	9.50	9.96	12.95	16.17

Supplementary Table 15 | Summary of the data shown in Supplementary Figure 17. Distribution of oligomer length after cycles 1, 2, 3, 5, and 10 in the solution-start samples prepared with Na_2HPO_4. The initial dry down of the stock solution was 6 hours at 100 °C. Each of the subsequent cycles was 18 hours at 40 °C and 50 %RH followed by 6 hours at 100 °C. Oligomers without entries for a particular cycle were not produced in a quantity greater than their limit of quantitation by our method of analysis. Source data are provided as a Source Data file.

	**Average Yield After Each Cycle (%)**				
Gly_n	Cycle 1	Cycle 2	Cycle 3	Cycle 5	Cycle 10
2	0.40	0.52	0.56	0.54	0.60
3	0.09	0.18	0.27	0.30	0.37
4		0.05	0.08	0.12	0.21
5		0.01	0.03	0.04	0.15
6				0.02	0.11
7				0.01	0.06
8					0.03
Total	0.49	0.75	0.93	1.03	1.54

Supplementary Table 16 | Summary of the data shown in Supplementary Figure 18. Distribution of oligomer length after cycles 1, 2, 3, 5, and 10 in the solution-start samples prepared with K_2HPO_4. The initial dry down of the stock solution was 4 hours at 120 °C. Each of the subsequent cycles was 20 hours at 40 °C and 50 %RH followed by 4 hours at 120 °C. Oligomers without entries for a particular cycle were not produced in a quantity greater than their limit of quantitation by our method of analysis. Source data are provided as a Source Data file.

	**Average Yield After Each Cycle (%)**				
Gly_n	Cycle 1	Cycle 2	Cycle 3	Cycle 5	Cycle 10
2	6.85	11.09	12.50	13.09	12.23
3	3.25	3.86	4.97	5.45	4.53
4	0.65	0.82	1.28	1.45	1.29
5	0.22	0.27	0.50	0.64	0.70
6	0.06	0.08	0.20	0.27	0.42
7	0.03	0.04	0.07	0.15	0.37
8			0.04	0.07	0.22
9				0.05	0.11
10					0.04
Total	11.07	16.16	19.57	21.16	19.93

Supplementary Table 17 | Summary of the data shown in Supplementary Figure 19. Distribution of oligomer length after cycles 1, 2, 3, 5, and 10 in the solution-start samples prepared with Na_2HPO_4. The initial dry down of the stock solution was 4 hours at 120 °C. Each of the subsequent cycles was 20 hours at 40 °C and 50 %RH followed by 4 hours at 120 °C. Oligomers without entries for a particular cycle were not produced in a quantity greater than their limit of quantitation by our method of analysis. Source data are provided as a Source Data file.

	**Average Yield After Each Cycle (%)**				
Gly_n	Cycle 1	Cycle 2	Cycle 3	Cycle 5	Cycle 10
2	0.83	0.70	0.83	0.75	0.84
3	0.34	0.48	0.64	0.58	0.60
4	0.08	0.20	0.31	0.32	0.40
5	0.03	0.11	0.22	0.26	0.34
6		0.06	0.16	0.20	0.31
7		0.03	0.09	0.13	0.22
8		0.01	0.05	0.07	0.13
9			0.03	0.04	0.07
10			0.01	0.02	0.05
11				0.01	0.02
12					0.01
Total	1.27	1.59	2.33	2.36	2.99

Supplementary Table 18 | Summary of the data shown in Supplementary Figure 21. Distribution of oligomer length after cycles 1, 2, 3, 5, and 10 in the samples prepared with KH_2PO_4. Each cycle was 18 hours at 40 °C and 70 %RH followed by 6 hours at 100 °C. Oligomers without entries for a particular cycle were not produced in a quantity greater than their limit of quantitation by our method of analysis. Source data are provided as a Source Data file.

	Average Yield After Each Cycle (%)				
Gly_n	Cycle 1	Cycle 2	Cycle 3	Cycle 5	Cycle 10
2	0.01	0.01	0.01	0.01	0.02
Total	0.01	0.01	0.01	0.01	0.02

Supplementary Table 19 | Summary of the data shown in Supplementary Figure 22. Distribution of oligomer length after cycles 1, 2, 3, 5, and 10 in the samples prepared with NaH_2PO_4. Each cycle was 18 hours at 40 °C and 70 %RH followed by 6 hours at 100 °C. Oligomers without entries for a particular cycle were not produced in a quantity greater than their limit of quantitation by our method of analysis. Source data are provided as a Source Data file.

	Average Yield After Each Cycle (%)				
Gly_n	Cycle 1	Cycle 2	Cycle 3	Cycle 5	Cycle 10
2	0.90	1.09	1.94	2.91	4.32
3	0.04	0.05	0.14	0.44	1.51
4			0.01	0.11	0.33
5					0.14
Total	0.94	1.14	2.09	3.46	6.30

Supplementary Table 20 | Summary of the data shown in Supplementary Figure 24. Distribution of oligomer length after cycles 1, 2, 3, 5, and 10 in the samples prepared with KBr. Each cycle was 18 hours at 40 °C and 50 %RH followed by 6 hours at 100 °C. Oligomers without entries for a particular cycle were not produced in a quantity greater than their limit of quantitation by our method of analysis. Source data are provided as a Source Data file.

	Average Yield After Each Cycle (%)				
Gly_n	Cycle 1	Cycle 2	Cycle 3	Cycle 5	Cycle 10
2	0.88	1.04	1.64	1.25	1.85
3	0.21	0.21	0.38	0.32	0.66
4		0.07	0.04	0.02	0.10
Total	1.09	1.32	2.06	1.59	2.61

Supplementary Table 21 | Summary of the data shown in Supplementary Figure 25. Distribution of oligomer length after cycles 1, 2, 3, 5, and 10 in the samples prepared with NaBr. Each cycle was 18 hours at 40 °C and 50 %RH followed by 6 hours at 100 °C. Oligomers without entries for a particular cycle were not produced in a quantity greater than their limit of quantitation by our method of analysis. Source data are provided as a Source Data file.

	**Average Yield After Each Cycle (%)**				
Gly_n	Cycle 1	Cycle 2	Cycle 3	Cycle 5	Cycle 10
2	1.18	1.35	2.37	3.50	5.79
3	0.31	0.39	0.66	1.52	3.55
4	0.05	0.06	0.10	0.26	0.85
5				0.06	0.32
6					0.08
7					0.03
Total	1.53	1.81	3.13	5.34	10.62

Supplementary Table 22 | Summary of the data shown in Supplementary Figure 26. Distribution of oligomer length after cycles 1, 2, 3, 5, and 10 in the samples prepared with KBr. Each cycle was 18 hours at 40 °C and 70 %RH followed by 6 hours at 100 °C. Oligomers without entries for a particular cycle were not produced in a quantity greater than their limit of quantitation by our method of analysis. Source data are provided as a Source Data file.

	**Average Yield After Each Cycle (%)**				
Gly_n	Cycle 1	Cycle 2	Cycle 3	Cycle 5	Cycle 10
2	1.41	2.47	2.93	4.39	7.31
3	0.23	0.61	0.80	1.96	3.35
4		0.06	0.07	0.25	0.68
5				0.07	0.25
6					0.01
Total	1.64	3.14	3.80	6.66	11.61

Supplementary Table 23 | Summary of the data shown in Supplementary Figure 27. Distribution of oligomer length after cycles 1, 2, 3, 5, and 10 in the samples prepared with NaBr. Each cycle was 18 hours at 40 °C and 70 %RH followed by 6 hours at 100 °C. Oligomers without entries for a particular cycle were not produced in a quantity greater than their limit of quantitation by our method of analysis. Source data are provided as a Source Data file.

	**Average Yield After Each Cycle (%)**				
Gly_n	Cycle 1	Cycle 2	Cycle 3	Cycle 5	Cycle 10
2	2.53	2.53	4.77	7.07	7.20
3	0.45	0.57	1.82	3.51	3.95
4	0.05	0.07	0.26	0.63	0.90
5			0.06	0.19	0.30
6					0.08
Total	3.03	3.16	6.92	11.40	12.43

Supplementary Table 24 | MALDI data of glycine oligomerization reaction after cycle 15. The samples were prepared with $(Na^+,K^+)(Cl^-,OH^-)$. The MALDI spectra are shown in Supplementary Figure 30. Each cycle was 20 hours at 40 °C and 70 %RH followed by 4 hours at 120 °C.

**Gly_n Oligomer**	**Theoretical $[M+Na]^+$**	**Experimental $[M+Na]^+$**	**Mass Error (ppm)**	**S/N**	
2	155.043	N/A	N/A	N/A	(below m/z range)
3	212.065	212.051	−66.0	44.1	
4	269.086	269.073	−48.3	21.0	
5	326.107	326.087	−61.3	11.0	
6	383.129	383.106	−60.0	19.6	
7	440.150	440.180	68.2	61.4	
8	497.172	497.189	34.2	59.5	
9	554.193	554.192	− 1.8	34.9	
10	611.214	611.218	6.5	16.1	
11	668.236	668.272	53.9	8.5	

Supplementary Table 25 | ICP–OES data. Ion concentrations obtained by ICP–OES analysis of the closed-capped samples for the (Na^+,K^+)(Cl^-,OH^-) system. Each cycle was 18 hours at 40 °C and 70 %RH followed by 6 hours at 100 °C.

	**[Na^+] (ppm)**	**[Na^+] (M)**	**[K^+] (ppm)**	**[K^+] (M)**	**Na^+:K^+**
**Cycle 1**	100	4.3	58	1.5	2.9 : 1
**Cycle 2**	86	3.7	51	1.3	2.9 : 1
**Cycle 3**	91	4.0	53	1.4	2.9 : 1
**Cycle 10**	88	3.8	53	1.4	2.8 : 1

Supplementary Methods

General considerations. All research chemicals were purchased as reagent grade and used without further purification. Glycine was purchased from TCI America (Portland, Oregon). The chloride, hydroxide, bromide, and phosphate salts of potassium and sodium were purchased from Flinn Scientific (Batavia, Illinois), MilliporeSigma (Burlington, Massachusetts), or VWR International (Radnor, Pennsylvania) and their affiliate suppliers.

Massing of deliquescent salts. The solid samples of glycine and salts used in this study were stored and massed in either a small room or in a tent (constructed from PVC and plastic sheeting) equipped with a residential-grade dehumidifier (Hisense DH-70KP1SLE). This precaution was taken to minimize the amount of moisture taken up by the solids while being handled. The goal was to maintain an atmosphere of room temperature (approximately 23 °C) and 25% relative humidity or lower.

Selection of the composition of the deliquescent mixtures. Given our desire to work with the chloride salts of potassium and sodium for their abundance on Earth and presumed prebiotic relevance, we first assessed the ability of mixtures of K^+ and Na^+ chloride and hydroxide salts to deliquesce at 40 °C, 70 %RH. We combined 1 M solutions of KCl, NaCl,

KOH, and NaOH into 2 mL mixtures in various ratios by micropipette. We heated all samples to dryness, then placed them into a humidity chamber set to 40 °C and 70 %RH. Each sample was massed before and after 24 hours in the humidity chamber. We used these data to construct Supplementary Fig. 3, which plots the ratio of the moles of water gained by the sample to the total moles of salt combined in the sample as a function of the ratio of Na^{+}:K^{+} in the sample. We selected a Na:K ratio of 3:1 (25% K^{+}) for the subsequent cycling experiments based on its propensity to deliquesce, as judged by having gained the most water mass at 70 %RH relative to other mixtures. The 3:1 ratio was preserved throughout the cycling experiments, as determined by ICP–OES analysis of the closed-capped samples (Supplementary Table 25) in the experiment described on page 41 of the Supplementary Information.

Procedure for solution-start control experiments. Most of the deliquescence experiments were started from a dry state, as described in the procedure in the main manuscript. As controls, we also conducted cycled reactions where the mixtures began as homogeneous solutions. In these experiments, solutions of 2:1 molar ratio glycine:total salt were mixed in 50 mL deionized water (Milli-Q® 18.2 MΩ·cm). The solutions were transferred to scintillation vials in 500 µL portions. For the first cycle, the samples were dried for 6 hours at 100 °C on the hot plates. There was no wet phase for the first cycle because the samples started as aqueous solutions. The subsequent cycles consisted of wet phases at 18 hours at 40 °C and 50 %RH (maintained by a humidity-controlled chamber) followed by 6 hours at 100 °C on a hot plate.

Notes on reporting yields of oligomers. We measured the yield of individual oligomers of glycine using the method we previously described for the quantitative analysis of glycine oligomerization.[1] Supplementary Figs 4–11 and Supplementary Tables 2–9 show the individual yields for each oligomer detected using IP–HPLC for each cycle for the (K^{+},Na^{+})(Cl^{-},OH^{-})

samples at 30–70 %RH and the $(K/Na)_2HPO_4$ samples at 50 %RH. Note that in some cases, the total yield of oligomers—defined as the percent of glycine starting material converted into any linear oligomers (Gly_n, n≥2, not including DKP)—may be unchanged or decreasing from one cycle to the next, but the yield of longer oligomers is increasing. This is particularly apparent in Supplementary Fig. 10 (K_2HPO_4 samples). Supplementary Figs 12–19 and Supplementary Tables 10–15 show the yield comparisons and the individual yields for glycine oligomers from the solution-start cycling experiments of (K,Na)(Cl,OH) and $(K/Na)_2HPO_4$ samples. Supplementary Figs 20–27 and Supplementary Tables 16–21 show the yield comparisons and the individual yields for glycine oligomers from control experiments of (K,Na)(Cl,OH) and $(K/Na)H_2PO_4$ samples. As noted in the main paper, we could not analyze any insoluble precipitates produced in the reactions by IP–HPLC. Given that these could be higher oligomers of Gly_n,[2] we may be reporting artificially low yields in a few systems. The reactions that produced observable solid precipitates were: the (Na,K)(Cl,OH) samples for cycle 10 at 70 %RH, 120 °C and the K_2HPO_4 samples for cycle 10 at 50 %RH, 120 °C. We sometimes observed solids in the cycle 5 samples for those two systems and the cycle 10 samples for (Na,K)(Cl,OH) at 100 °C, but their production was not consistent.

Analysis by MALDI–TOF mass spectrometry. The structural similarity of the individual glycine oligomers precludes their analysis by spectroscopic methods, like NMR spectroscopy, and can make resolution difficult in chromatographic methods. We pursued MALDI–TOF mass spectrometry as a method to corroborate our analysis by IP–HPLC and improve upon its shortcomings (e.g., the limits of detection of higher oligomers). We had limited success, presumably due to the large quantity of salts in our samples. An IP–HPLC chromatogram (from our existing method of analysis) and MALDI–TOF mass spectrum of an

identical sample are juxtaposed as Supplementary Figs 29 and 30, respectively. The MS data are listed in Supplementary Table 22. It is promising that all of the oligomers observed by IP–HPLC in this sample (specifically, $Gly_{3–11}$) are observed in the mass spectrum as sodium adducts; however, the MALDI spectrum is dominated by salt cluster peaks. Consequently, in the main manuscript, we have limited the analysis presented to the more robust IP–HPLC method.

Current protocol for MALDI–TOF analysis. Samples were dissolved in deionized water with 0.1% trifluoroacetic acid (TFA) and centrifuged at 5000 rpm for 2 minutes. The supernatant was discarded and the pellets redissolved in 200 µL of 50% deionized water and 50% acetonitrile with 0.1% TFA. This mixture was vortexed then sonicated for 10 minutes. We spotted 1.0 µL of sample and 1.0 µL of 10 mg/mL 2,5-dihydroxybenzoic acid (DHB) matrix. The target plate was left to evaporate and then loaded into a Voyager DE-STR time-of-flight mass spectrometer. The TOF was calibrated with the following signals: DHB matrix dimer, bradykinin 1-5, bradykinin, angiotensin I, neurotensin. Signal-to-noise (S/N) values were calculated using Data Explorer 4.0 software. The instrument settings were the following: positive ion mode; 200–2000 m/z range; 20000 V acceleration voltage; reflector TOF mode; 76% extraction grid voltage; 150 ns extraction delay time; 100 shots per spectrum; 2015 (arbitrary units) laser attenuation; 20 Hz laser repetition rate; 337 nm (N_2) laser wavelength.

Summary of protocol optimization for MALDI–TOF analysis. Briefly, several approaches were tried before settling on the protocol above. Initially, samples were dissolved in 50% water / 50% acetonitrile and were spotted on top of matrix without any purification; however, very few analytes were observed due to salt suppression. Second, samples were dissolved in 0.1% TFA and were then subjected to an on-plate water wash to reduce the salt present. Again, few Gly_n signals were detected with very low signal-to-noise (S/N). The third

approach, known as the fast evaporation method,[3] consisted of dissolving matrix in acetone such that the matrix dried rapidly on the target, forming a thin hydrophobic layer of crystals. The sample was dissolved in 0.1% TFA in water and was spotted on top of the matrix in order to extract the oligomers onto the matrix layer. This too did not produce sufficient analyte S/N.

Procedure for excess rehydration experiments. The (K/Na)(Cl/OH)-glycine stock mixture described in the main paper was transferred to scintillation vials in 50 mg portions. Each reaction was run in triplicate, and each run required five vials per reaction mixture, as one vial was removed for analysis at the end of cycles 1, 2, 3, 5, and 10 similar to the standard experiments. For the wet phase of each cycle, the vials were placed in a temperature and humidity-controlled chamber (an FWE Clymate IQ® cabinet) set to 40 °C and 70 %RH. For the dry phase of each cycle, the samples were transferred to a hot plate set at 100 °C for 6 hours. As opposed to the standard experiments, 20 mL of water was added to the mixture before each drying period to simulate heavy rain and overhydration. The samples were analyzed at the conclusion of the drying portion of cycles by IP–HPLC.

Procedure for closed-capped brine rehydration experiments. To assess how water activity affects our deliquescent systems, a 5 g portion of the (K^+/Na^+)($^-$Cl/$^-$OH)-glycine stock mixture described in the main paper was transferred to a high-pressure glass vial (Chemglass Life Sciences LLC, Vineland, NJ). The sample was let to deliquesce for 5 days in a humidity-controlled chamber set to 40 °C and 70 %RH (~7.5 g of water was sequestered at equilibrium, as determined by the increase in mass of the sample). The sample was capped and subjected to cycles of 100 °C for 6 hours while immersed in a sand bath followed by 18 hours at 40 °C. At the conclusion of hot phases for cycles 1, 2, 3, 5, and 10, 100 μL aliquots were transferred to microcentrifuge tubes for analysis by IP–HPLC and ICP–OES.

Density measurements of the deliquescent brine. At the conclusion of the cycling experiment of the closed-capped system, three 800 μL aliquots of the sample were massed on an analytical balance (a Sartorius Practum 124–1S) to measure the density of the brine. The density was determined to be 1.33 ± 0.02 g/mL (n=3 identical experiments).

Procedure for excess hydration with brine. As a control, the $(K^+/Na^+)(^-Cl/^-OH)$-glycine stock mixture described in the main paper was transferred to scintillation vials in 50 mg portions. The reaction was run in triplicate, and each run required five vials per reaction mixture, as one vial was removed for analysis at the end of cycles 1, 2, 3, 5, and 10, similar to the standard experiments. As opposed to the experiments described above, 20 mL of a solution saturated in the $(K^+/Na^+)(^-Cl/^-OH)$ salt mixture was added to the reaction before the first drying period. For the wet phase of each cycle, the vials were placed in a temperature and humidity-controlled chamber (an FWE Clymate IQ® cabinet) set to 40 °C and 70 %RH. For the dry phase of each cycle, the samples were transferred to a hot plate set at 100 °C for 6 hours. At the conclusion of the last dry cycle, we removed the excess water from the system by vacuum evaporation using a Labconco CentriVap® vacuum concentrator at 45 °C under 5 Torr overnight. The samples were analyzed at the conclusion of the drying portion of cycles by IP–HPLC.

Inductively-coupled plasma–optical-emission spectroscopy. A 10 μL aliquot of each sample was diluted to 10 mL with 2% HNO_3. Samples were measured for sodium and potassium concentrations using a Perkin–Elmer Optima 8300 ICP Spectrometer. The instrument is equipped with an Elemental Scientific (ESI) prep*FAST* autosampler system and utilizes a cross-flow nebulizer with the following parameters: plasma 10 $Ar_{(g)}$ L min^{-1}; auxiliary 0.2 $Ar_{(g)}$ L

min^{-1}; nebulizer 0.65 $Ar_{(g)}$ L min^{-1}; power 1,400 W. Calibration curves constructed for Na^+ and K^+ using a 10 mg L^{-1} elemental solutions (Sigma–Aldrich) resulted in ≤2% error.

Supplementary Discussion

The downturn in total yield at higher temperatures. We speculate that the downturn in total yield sometimes observed at higher temperatures at higher cycles—such as between the fifth and tenth cycles at 120 °C in Figure 2b—could be attributable to the production of oligomers longer than Gly_{14}, diketopiperazine (DKP), or unknown decomposition products. Consistent with previous reports, we sometimes noted the formation of white precipitate—presumably insoluble oligomers of $Gly_{>14}$—that could not be analysed by our IP–HPLC method.[2] The apparent downturn could be due to our inability to measure products longer than Gly_{14} rather than a decreased conversion of glycine to oligomer products. Alternate possible explanations for the downturn include thermal degradation of glycine and its oligomers,[2, 4] as the reaction mixtures had a tendency to discolor (from white to pale yellow) with increasing time and heat, again consistent with previous reports of heated mixtures of glycine.[2, 5] Finally, it is possible that the conditions favored the formation of DKP—cyclic Gly_2—which also could not be measured by our IP–HPLC method.

Effect of water activity. We conducted two modified cycling experiments to test the effect of water activity in these systems. First, we performed a standard cycling experiment with the modification of not evaporating water from the samples during heating periods. We allowed the initial reaction mixture of glycine in the (K^+,Na^+)(^-Cl,OH^-) salt mixture to acquire water over 5 days at 40° C and 70 %RH. We then placed this mixture in a capped tube and subjected it to cycled phases of 6 h at 100 °C and 18 h at 40 °C. The solution did not evaporate because it was

capped, but we still observed the formation of some Gly_2 and Gly_3 despite the presence of water and the possibility of hydrolysis. The results are plotted in Supplementary Fig. 31, as a green line added to the plot that appears as Figure 3 in the main paper. This experiment suggests the water activity in the system is low enough to support the formation of some condensation product, but relative to the lower water activity afforded by evaporating to dryness, the yields and lengths of the oligomers are lower.

We also modified the overhydration experiment plotted in Figure 3 of the main paper by rehydrating with brine rather than deionized water. This experiment simulates an evaporating pond that is periodically refilled by ocean water. We expected to add 20 mL of brine after each cycle, but the amount of salt present led to precipitation during the first drying phase that precluded adding more brine after the first addition. A cap of precipitated salt formed at the top of the solution and prevented evaporation to dryness (see photograph in Supplementary Fig. 32). We continued to heat (6 h at 100 °C) and cool (6 h at 100 °C & 70 %RH) the samples for ten cycles, but we did not observe the formation of any oligomers by IP–HPLC. For this system, it appears the water activity is high enough to favor hydrolysis over condensation, though we hesitate to draw firm conclusions without further experimentation.

Summary of results. The figures and tables that compose the majority of this document can look similar such that it is difficult to decipher the importance of any individual experiment. In this section, we begin by organizing these data in tables and summarize the take-home messages from the experiments to complement the discussion in the text of the main paper.

The top-level perspective provided by Supplementary Table 1 makes clear that of all the factors tested, whether or not a sample is deliquescent has the major impact on total yield. In the chloride systems, where the reaction mixtures are identical, the samples rehydrated at 70 %RH

(where the mixtures are deliquescent) give much higher yields than those rehydrated at 30 and 50 %RH (below the threshold of RH_0 for deliquescence). In the bromide and phosphate systems, the reaction mixtures were not identical; they differed in the presence of K^+ or Na^+ as the countercation. But in these systems as well, higher yields were always generated in the samples that were deliquescent. Sometimes the deliquescent member of paired samples occurred when K^+ was the cation (e.g., bromides and dibasic phosphates); sometimes the deliquescent member of the pair occurred when Na^+ was the cation (e.g., bromides and monobasic phosphates). Higher temperatures during the dry phase and higher humidities during the wet phase had only modest effects on yields when both samples were deliquescent in the wet phase (e.g., for the sodium bromide samples).

The effect of proton activity—a term we prefer over pH when discussing reaction mixtures where the temperature and activity of water vary so widely—can be seen by contrasting the results for the monobasic and dibasic phosphates. In both cases, the deliquescent mixtures (K_2HPO_4 and NaH_2PO_4) gave higher yields than their non-deliquescent counterparts paired with the other alkali ion. But the yield in K_2HPO_4 was significantly higher than for NaH_2PO_4, consistent with the condensation being catalyzed by base. Still, peptide formation mediated by deliquescent wet–dry cycling does occur under mildly acidic conditions, which is notable considering the presumed increased acidity of the Prebiotic Earth.[6] The conditions used in our main system, (K^+/Na^+)($^-Cl/^-OH$), are strongly basic. Alkaline conditions and hydroxide/oxide minerals have been proposed to exist on the early Earth in hydrothermal environments and those subjected to metamorphism and other geological processes.[7, 8]

Supplementary Table 2 summarizes the results for experiments where the reaction mixtures began as homogeneous aqueous solutions (instead of as solid mixtures, in

Supplementary Table 1). In these experiments, the first cycle was always wet, even for mixtures that were not deliquescent. The mixtures that were deliquescent at the %RH of the cool cycles rehydrated during each cycle, while those that were not deliquescent remained as solids throughout cycles 2 through 10.

These experiments were principally controls that demonstrated there was no major difference in yield whether the reagents were mixed directly as solids or as solutions. Whether or not a mixture is deliquescent is still the major factor determining total yield. However, we did observe that starting as a solution (vs. solid) did confer an advantage to mixtures that were not deliquescent: even though the yields were low, they produced longer oligomers than the corresponding solid-start experiments in Supplementary Table 1.

Context of our perceived significance of the results. In this paper, we report that mixtures of prebiotically and biologically relevant deliquescent salts can serve as media to host wet–dry cycles and self-regulate the moisture content and/or volume of a system. Wet–dry cycles have been used many times in the past as an important means to drive a variety of prebiotically relevant condensation reactions.[2, 9-17]

Note that the yields we report are lower than in some previously reported systems,[2] but the yields are commensurate with the shortened drying time in our system. The truncated drying times are justifiable on the grounds of both experimental design and prebiotic relevance. By shortening the drying time for one cycle to just 4–6 hours, we can observe changes from one cycle to the next, and evaluate the importance of cycling and the value of the deliquescent properties of the sample. Furthermore, on the Prebiotic Earth, short periods of drying that match the diurnal cycle seem more likely than sustained temperatures over 100 °C.

It is difficult to imagine a location on early Earth that would be reliably subjected to a single daily rainstorm, let alone storms of regular volume. Rather, it seems likely that storms would not always be predictable in schedule or duration, such that reaction mixtures would need the ability to tolerate extra water and the occasional flood. In contrast, there are extant environments on Earth and Mars where oscillations in humidity are known to cause phase changes of deliquescent minerals. Collectively, the experiments here demonstrate the importance of regulated rehydration in wet–dry cycling models. Both over- and under-hydration of the reaction mixtures create problems. Previous experiments have been able to optimize the rehydration with human intervention, but deliquescence offers a natural mechanism for achieving a sweet spot of rehydration.

Future directions. This paper is a communication of our initial results with respect to deliquescent wet–dry cycling. We have a number of follow-up experiments currently planned and in progress. We are particularly interested in what other substrates can undergo condensation to form prospective protobiopolymers, which includes expanding our studies to amino acids other than glycine. We are also interested in surveying other deliquescent minerals for their effects on yields in these systems, including sulfate and carbonate salts.[7]

We remain interested in investigating the effects of water activity and what chemistry is supported at various points along these cycles. Studies of the kinetics and thermodynamics of condensation and hydrolysis will be of critical importance in any thorough evaluation of these systems. The effects of the inclusion of prebiotically relevant catalysts, such as transition metals and clay minerals, is also of great interest to us. Some clay and salt minerals have been shown to catalyze the formation of potential protobiopolymers and protocellular compartments, including through condensation mediated by wet–dry cycling.[13, 18, 19] We reiterate that this communication

is an initial report of a novel system. We will continue to test it, and we encourage others to do so as well.

Supplementary References

1. Campbell, T. D., Febrian, R., Kleinschmidt, H. E., Smith, K. A., Bracher, P. J. Quantitative analysis of glycine oligomerization by ion-pair chromatography. *ACS Omega*, **4**, 12745-12752 (2019).

2. Rodriguez-Garcia, M., et al. Formation of oligopeptides in high yield under simple programmable conditions. *Nat. Commun.*, **6**, doi: 10.1038/ncomms9385 (2015).

3. Vorm, O., Roepstorff, P., Mann, M. Improved resolution and very high sensitivity in MALDI TOF of matrix surfaces made by fast evaporation. *Anal. Chem.*, **66**, 3281-3287 (1994).

4. Weiss, I. M., Muth, C., Drumm, R., Kirchner, H. O. K. Thermal decomposition of the amino acids glycine, cysteine, aspartic acid, asparagine, glutamic acid, glutamine, arginine and histidine. *BMC Biophysics*, **11**, doi: 10.1186/s13628-13018-10042-13624 (2018).

5. Ohara, S., Kakegawa, T., Nakazawa, H. Pressure effects on the abiotic polymerization of glycine. *Orig. Life Evol. Biosph.*, **37**, 215-223 (2007).

6. Krissansen-Totton, J., Arney, G. N., Catling, D. C. Constraining the climate and ocean pH of the early Earth with a geological carbon cycle model. *Proc. Natl. Acad. Sci. U.S.A.*, **115**, 4105-4110 (2018).

7. Hazen, R. M. Paleomineralogy of the hadean eon: a preliminary species list. *Am. J. Sci.*, **313**, 807-843 (2013).

8. Russell, M. J., et al. The drive to life on wet and icy worlds. *Astrobiology*, **14**, 308-343 (2014).

9. Becker, S., et al. Wet-dry cycles enable the parallel origin of canonical and non-canonical nucleosides by continuous synthesis. *Nat. Commun.*, **9**, doi: 10.1038/s41467-41017-02639-41461 (2018).

10. Da Silva, L., Maurel, M. C., Deamer, D. Salt-promoted synthesis of RNA-like molecules in simulated hydrothermal conditions. *J. Mol. Evol.*, **80**, 86-97 (2015).

11. Forsythe, J. G., et al. Ester-mediated amide bond formation driven by wet-dry cycles: a possible path to polypeptides on the prebiotic Earth. *Angew. Chem. Int. Ed.*, **54**, 9871-9875 (2015).

12. Fox, S. W., Harada, K. The thermal copolymerization of amino acids common to protein. *J. Am. Chem. Soc.*, **82**, 3745-3751 (1960).

13. Lahav, N., White, D., Chang, S. Peptide formation in the prebiotic era: thermal condensation of glycine in fluctuating clay environments. *Science*, **201**, 67-69 (1978).

14. Mamajanov, I., et al. Ester formation and hydrolysis during wet-dry cycles: generation of far-from-equilibrium polymers in a model prebiotic reaction. *Macromolecules*, **47**, 1334-1343 (2014).

15. Nelson, K. E., Robertson, M. P., Levy, M., Miller, S. L. Concentration by evaporation and the prebiotic synthesis of cytosine. *Orig. Life Evol. Biosph.*, **31**, 221-229 (2001).

16. Rajamani, S., Vlassov, A., Benner, S., Coombs, A., Olasagasti, F., Deamer, D. Lipid-assisted synthesis of RNA-like polymers from mononucleotides. *Orig. Life Evol. Biosph.*, **38**, 57-74 (2008).

17. Shen, C., Lazcano, A., Oro, J. The enhancement activities of histidyl-histidine in some prebiotic reactions. *J. Mol. Evol.*, **31**, 445-452 (1990).

18. Zhou, R., et al. Catalyzed synthesis of zinc clays by prebiotic central metabolites. *Sci. Rep.*, **7**, doi: 10.1038/s41598-41017-00558-41591 (2017).

19. Hanczyc, M. M., Fujikawa, S. M., Szostak, J. W. Experimental models of primitive cellular compartments: encapsulation, growth, and division. *Science*, **302**, 618-622 (2003).
